# FinnGen provides genetic insights from a well-phenotyped isolated population

**DOI:** 10.1038/s41586-022-05473-8

**Published:** 2023-01-18

**Authors:** Mitja I. Kurki, Juha Karjalainen, Priit Palta, Timo P. Sipilä, Kati Kristiansson, Kati M. Donner, Mary P. Reeve, Hannele Laivuori, Mervi Aavikko, Mari A. Kaunisto, Anu Loukola, Elisa Lahtela, Hannele Mattsson, Päivi Laiho, Pietro Della Briotta Parolo, Arto A. Lehisto, Masahiro Kanai, Nina Mars, Joel Rämö, Tuomo Kiiskinen, Henrike O. Heyne, Kumar Veerapen, Sina Rüeger, Susanna Lemmelä, Wei Zhou, Sanni Ruotsalainen, Kalle Pärn, Tero Hiekkalinna, Sami Koskelainen, Teemu Paajanen, Vincent Llorens, Javier Gracia-Tabuenca, Harri Siirtola, Kadri Reis, Abdelrahman G. Elnahas, Benjamin Sun, Christopher N. Foley, Katriina Aalto-Setälä, Kaur Alasoo, Mikko Arvas, Kirsi Auro, Shameek Biswas, Argyro Bizaki-Vallaskangas, Olli Carpen, Chia-Yen Chen, Oluwaseun A. Dada, Zhihao Ding, Margaret G. Ehm, Kari Eklund, Martti Färkkilä, Hilary Finucane, Andrea Ganna, Awaisa Ghazal, Robert R. Graham, Eric M. Green, Antti Hakanen, Marco Hautalahti, Åsa K. Hedman, Mikko Hiltunen, Reetta Hinttala, Iiris Hovatta, Xinli Hu, Adriana Huertas-Vazquez, Laura Huilaja, Julie Hunkapiller, Howard Jacob, Jan-Nygaard Jensen, Heikki Joensuu, Sally John, Valtteri Julkunen, Marc Jung, Juhani Junttila, Kai Kaarniranta, Mika Kähönen, Risto Kajanne, Lila Kallio, Reetta Kälviäinen, Jaakko Kaprio, FinnGen, Nurlan Kerimov, Johannes Kettunen, Elina Kilpeläinen, Terhi Kilpi, Katherine Klinger, Veli-Matti Kosma, Teijo Kuopio, Venla Kurra, Triin Laisk, Jari Laukkanen, Nathan Lawless, Aoxing Liu, Simonne Longerich, Reedik Mägi, Johanna Mäkelä, Antti Mäkitie, Anders Malarstig, Arto Mannermaa, Joseph Maranville, Athena Matakidou, Tuomo Meretoja, Sahar V. Mozaffari, Mari E. K. Niemi, Marianna Niemi, Teemu Niiranen, Christopher J. O´Donnell, Ma´en Obeidat, George Okafo, Hanna M. Ollila, Antti Palomäki, Tuula Palotie, Jukka Partanen, Dirk S. Paul, Margit Pelkonen, Rion K. Pendergrass, Slavé Petrovski, Anne Pitkäranta, Adam Platt, David Pulford, Eero Punkka, Pirkko Pussinen, Neha Raghavan, Fedik Rahimov, Deepak Rajpal, Nicole A. Renaud, Bridget Riley-Gillis, Rodosthenis Rodosthenous, Elmo Saarentaus, Aino Salminen, Eveliina Salminen, Veikko Salomaa, Johanna Schleutker, Raisa Serpi, Huei-yi Shen, Richard Siegel, Kaisa Silander, Sanna Siltanen, Sirpa Soini, Hilkka Soininen, Jae Hoon Sul, Ioanna Tachmazidou, Kaisa Tasanen, Pentti Tienari, Sanna Toppila-Salmi, Taru Tukiainen, Tiinamaija Tuomi, Joni A. Turunen, Jacob C. Ulirsch, Felix Vaura, Petri Virolainen, Jeffrey Waring, Dawn Waterworth, Robert Yang, Mari Nelis, Anu Reigo, Andres Metspalu, Lili Milani, Tõnu Esko, Caroline Fox, Aki S. Havulinna, Markus Perola, Samuli Ripatti, Anu Jalanko, Tarja Laitinen, Tomi P. Mäkelä, Robert Plenge, Mark McCarthy, Heiko Runz, Mark J. Daly, Aarno Palotie

**Affiliations:** 1 Institute for Molecular Medicine Finland (FIMM), Helsinki Institute of Life Science (HiLIFE), University of Helsinki, Helsinki, Finland; 2 Program in Medical and Population Genetics, Broad Institute of Harvard and MIT, Cambridge, MA USA; 3 Stanley Center for Psychiatric Research, Broad Institute of Harvard and MIT, Cambridge, MA USA; 4 Analytic and Translational Genetics Unit, Massachusetts General Hospital, Boston, MA USA; 5 Estonian Genome Centre, Institute of Genomics, University of Tartu, Tartu, Estonia; 6 Finnish Institute for Health and Welfare (THL), Helsinki, Finland; 7 Medical and Clinical Genetics, University of Helsinki and Helsinki University Hospital, Helsinki, Finland; 8 Department of Obstetrics and Gynecology, Tampere University Hospital, Tampere, Finland; 9 Faculty of Medicine and Health Technology, Center for Child, Adolescent and Maternal Health, University of Tampere, Tampere, Finland; 10 Helsinki Biobank, University of Helsinki and Hospital District of Helsinki and Uusimaa, Helsinki, Finland; 11 Department of Biomedical Informatics, Harvard Medical School, Boston, MA USA; 12 Digital Health Center, Hasso Plattner Institute for Digital Engineering, University of Potsdam Potsdam, Potsdam, Germany; 13 Hasso Plattner Institute for Digital Health at Mount Sinai, Department of Genetics and Genomic Sciences, Icahn School of Medicine at Mount Sinai, New York, NY USA; 14 TAUCHI Research Center, Faculty of Information Technology and Communication Sciences, Tampere University, Tampere, Finland; 15 Translational Biology, Research and Development, Biogen, Cambridge, MA USA; 16 BHF Cardiovascular Epidemiology Unit, Department of Public Health and Primary Care, University of Cambridge, Cambridge, UK; 17 Optima Partners, Edinburgh, UK; 18 MRC Biostatistics Unit, School of Clinical Medicine, University of Cambridge, Cambridge, UK; 19 Faculty of Medicine and Health Technology, Tampere University, Tampere, Finland; 20 Institute of Computer Science, University of Tartu, Tartu, Estonia; 21 Finnish Red Cross Blood Service, Helsinki, Finland; 22 GlaxoSmithKline, Espoo, Finland; 23 Bristol Myers Squibb, New York, NY USA; 24 Tampere University Hospital and Tampere University, Tampere, Finland; 25 Biogen, Cambridge, MA USA; 26 Boehringer Ingelheim, Ingelheim am Rhein, Germany; 27 GlaxoSmithKline, Collegeville, PA USA; 28 Division of Rheumatology, Department of Medicine, Helsinki University Central Hospital, Helsinki, Finland; 29 Orton Orthopedic Hospital, Helsinki, Finland; 30 Abdominal Center, Helsinki University Hospital, Helsinki University, Helsinki, Finland; 31 Maze Therapeutics, South San Francisco, CA USA; 32 Auria Biobank, University of Turku and Turku University Hospital, Turku, Finland; 33 FINBB, Finnish Biobank Cooperative, Helsinki, Finland; 34 Pfizer, New York, NY USA; 35 Department of Medicine, Karolinska Institute, Solna, Sweden; 36 Clinical Biobank Tampere, Tampere University and Tampere University Hospital, Tampere, Finland; 37 Medical Research Center Oulu and PEDEGO Research Unit, University of Oulu, Oulu, Finland; 38 Biocenter Oulu, University of Oulu, Oulu, Finland; 39 Oulu University Hospital, Oulu, Finland; 40 Department of Psychology and Logopedics, Faculty of Medicine, University of Helsinki, Helsinki, Finland; 41 SleepWell Research Program, Faculty of Medicine, University of Helsinki, Helsinki, Finland; 42 Merck & Co, Kenilworth, NJ USA; 43 PEDEGO Research Unit, University of Oulu, Oulu, Finland; 44 Department of Dermatology and Medical Research Center Oulu, Oulu University Hospital, Oulu, Finland; 45 Genentech, San Francisco, CA USA; 46 AbbVie, Chicago, IL USA; 47 Helsinki University Hospital and University of Helsinki, Helsinki, Finland; 48 Neuro Center, Neurology, Kuopio University Hospital, Kuopio, Finland; 49 Institute of Clinical Medicine–Neurology, University of Eastern Finland, Kuopio, Finland; 50 Northern Finland Biobank Borealis, University of Oulu, Northern Ostrobothnia Hospital District, Oulu, Finland; 51 Department of Ophthalmology, Kuopio University Hospital, Kuopio, Finland; 52 Department of Ophthalmology, Institute of Clinical Medicine, University of Eastern Finland, Kuopio, Finland; 53 Department of Clinical Physiology, Tampere University Hospital, Tampere, Finland; 54 Epilepsy Center, Kuopio University Hospital, Kuopio, Finland; 55 Department of Neurology, University of Eastern Finland, Kuopio, Finland; 56 Department of Public Health, University of Helsinki, Helsinki, Finland; 57 Computational Medicine, Center for Life Course Health Research, Faculty of Medicine, University of Oulu, Oulu, Finland; 58 Translational Sciences, Sanofi R&D, Framingham, MA USA; 59 Biobank of Eastern Finland, University of Eastern Finland, Kuopio, Finland; 60 Kuopio University Hospital, Kuopio, Finland; 61 Central Finland Biobank, Central Finland Health Care District, Jyväskylä, Finland; 62 Department of Clinical Genetics, Tampere University Hospital, Tampere, Finland; 63 Department of Clinical Genetics, Faculty of Medicine and Health Technology, Tampere, Finland; 64 Department of Medicine, Institute of Clinical Medicine, University of Eastern Finland, Kuopio, Finland; 65 FINBB, Finnish Biobank Cooperative, Turku, Finland; 66 Department of Otorhinolaryngology–Head and Neck Surgery, University of Helsinki, Helsinki, Finland; 67 Helsinki University Hospital, Helsinki, Finland; 68 Pfizer, Cambridge, MA USA; 69 Department of Medical Epidemiology and Biostatistics, Karolinska Institute, Solna, Sweden; 70 Centre for Genomics Research, Discovery Sciences, BioPharmaceuticals R&D, AstraZeneca, Cambridge, UK; 71 TAUCHI Research Center & Faculty of Medicine and Health Technology, Tampere University, Tampere, Finland; 72 Turku University Hospital and University of Turku, Turku, Finland; 73 Novartis Institutes for BioMedical Research, Cambridge, MA USA; 74 Anesthesia, Critical Care, and Pain Medicine, Massachusetts General Hospital, Boston, MA USA; 75 Department of Oral and Maxillofacial Diseases, Helsinki University Hospital, Helsinki, Finland; 76 Department of Oral and Maxillofacial Diseases, University of Helsinki, Helsinki, Finland; 77 Finnish Hematological Biobank, Helsinki, Finland; 78 Department of Pulmonary Diseases, Kuopio University Hospital, Kuopio, Finland; 79 Department of Otorhinolaryngology, Helsinki University Hospital and University of Helsinki, Helsinki, Finland; 80 Translational Science and Experimental Medicine, Research and Early Development, Respiratory and Immunology, BioPharmaceuticals R&D, AstraZeneca, Cambridge, UK; 81 GlaxoSmithKline, Stevenage, UK; 82 Department of Clinical Genetics, HUSLAB, HUS Diagnostic Center, University of Helsinki, Helsinki, Finland; 83 Novartis Institutes for BioMedical Research, Basel, Switzerland; 84 Finnish Clinical Biobank Tampere, Tampere University and Tampere University Hospital, Tampere, Finland; 85 Department of Neurology, Institute of Clinical Medicine, University of Eastern Finland, Kuopio, Finland; 86 Department of Neurology, Helsinki University Hospital, Helsinki, Finland; 87 Translational Immunology, Research Programs Unit, University of Helsinki, Helsinki, Finland; 88 Department of Allergy, Helsinki University Hospital and University of Helsinki, Helsinki, Finland; 89 Abdominal Center, Endocrinology, Helsinki University Hospital, Helsinki, Finland; 90 Folkhalsan Research Center, Helsinki, Finland; 91 Research Program of Clinical and Molecular Metabolism, University of Helsinki, Helsinki, Finland; 92 Eye Genetics Group, Folkhälsan Research Center, Helsinki, Finland; 93 University of Turku, Turku, Finland; 94 Janssen Research & Development, Spring House, PA USA; 95 Janssen Biotech, Beerse, Belgium; 96 Genomics Core Facility, Institute of Genomics, University of Tartu, Tartu, Estonia; 97 Helsinki Institute of Life Science (HiLIFE), University of Helsinki, Helsinki, Finland

**Keywords:** Genome-wide association studies, Genetics research, Genetic predisposition to disease, Rare variants

## Abstract

Population isolates such as those in Finland benefit genetic research because deleterious alleles are often concentrated on a small number of low-frequency variants (0.1% ≤ minor allele frequency < 5%). These variants survived the founding bottleneck rather than being distributed over a large number of ultrarare variants. Although this effect is well established in Mendelian genetics, its value in common disease genetics is less explored^1,2^. FinnGen aims to study the genome and national health register data of 500,000 Finnish individuals. Given the relatively high median age of participants (63 years) and the substantial fraction of hospital-based recruitment, FinnGen is enriched for disease end points. Here we analyse data from 224,737 participants from FinnGen and study 15 diseases that have previously been investigated in large genome-wide association studies (GWASs). We also include meta-analyses of biobank data from Estonia and the United Kingdom. We identified 30 new associations, primarily low-frequency variants, enriched in the Finnish population. A GWAS of 1,932 diseases also identified 2,733 genome-wide significant associations (893 phenome-wide significant (PWS), *P* < 2.6 × 10^–11^) at 2,496 (771 PWS) independent loci with 807 (247 PWS) end points. Among these, fine-mapping implicated 148 (73 PWS) coding variants associated with 83 (42 PWS) end points. Moreover, 91 (47 PWS) had an allele frequency of <5% in non-Finnish European individuals, of which 62 (32 PWS) were enriched by more than twofold in Finland. These findings demonstrate the power of bottlenecked populations to find entry points into the biology of common diseases through low-frequency, high impact variants.

## Abstract (web summary)

Genome-wide association studies of individuals from an isolated population (data from the Finnish biobank study FinnGen) and consequent meta-analyses facilitate the identification of previously unknown coding variant associations for both rare and common diseases.

## Main

Large biobank studies have become an important source of genetic discoveries. The FinnGen study aims to construct a resource that combines the power of nationwide biobanks, structured national healthcare data and a unique, isolated population. Owing to increased genetic drift, isolated populations with recent bottlenecks can have deleterious, disease-predisposing alleles at considerably higher frequencies than permitted by selection in larger and older outbred populations. Counterbalancing this enrichment of specific low-frequency alleles, the other consequence of a recent bottleneck is that isolated populations have considerably fewer rare variants overall^1,3^. As a result, isolated populations provide an opportunity to identify high-impact disease variants that are rare in other populations^1,2^. In Finland, a strong founding bottleneck occurred about 120 generations ago followed by rapid population expansion. This bottleneck effect has resulted in numerous strongly deleterious alleles that occur more frequently in Finland compared with other European populations. This is manifested in the Finnish Disease Heritage, a set of 36 mostly recessive diseases that are more prevalent in Finland than elsewhere in the world^4^. This population history (which facilitates the identification of low-frequency deleterious alleles) combined with longitudinal information from registers that record hospital in-patient and outpatient diagnoses, purchases of prescription medications and many other national health registries centrally collected for decades provides valuable opportunities for understanding the genetic basis of health and disease.

FinnGen is a public–private partnership research project that combines imputed genotype data generated from newly collected and legacy samples from Finnish biobanks and digital health record data from Finnish health registries (https://www.finngen.fi/en) with the aim to provide new insights into disease genetics. FinnGen includes 9 Finnish biobanks, research institutes, universities and university hospitals, 13 international pharmaceutical industry partners and the Finnish Biobank Cooperative (FINBB) in a pre-competitive partnership. As of August 2020 (release 5 described in this article), samples from 412,000 individuals have been collected and have been 224,737 analysed with the aim to have a cohort of 500,000 participants ( Supplementary Methods, section 2). The project utilizes data from the nationwide longitudinal health register collected since 1969 from every resident in Finland.

Here we describe the FinnGen project and its current genotype and phenotype content and highlight a series of genetic discoveries from the first data collection phase. In other articles, we describe more detailed studies that showcase different aspects of the rich data available from population registries. Here we first show that FinnGen register-based phenotypes are comparable to those used in disease-specific GWASs in 15 previously well-studied common diseases. We demonstrate the power of the combination of data from an isolated population and other registers to discover new low-frequency variant associations, even in previously well-studied diseases in which FinnGen has a much smaller number of cases than in published disease-specific GWASs. Finally, through a GWAS of 1,932 end points followed by statistical fine-mapping, we demonstrate the ability to identify probable causal coding variants even with low allele frequencies (AFs).

## Phenotyping and genotyping

**Fig. 1:**
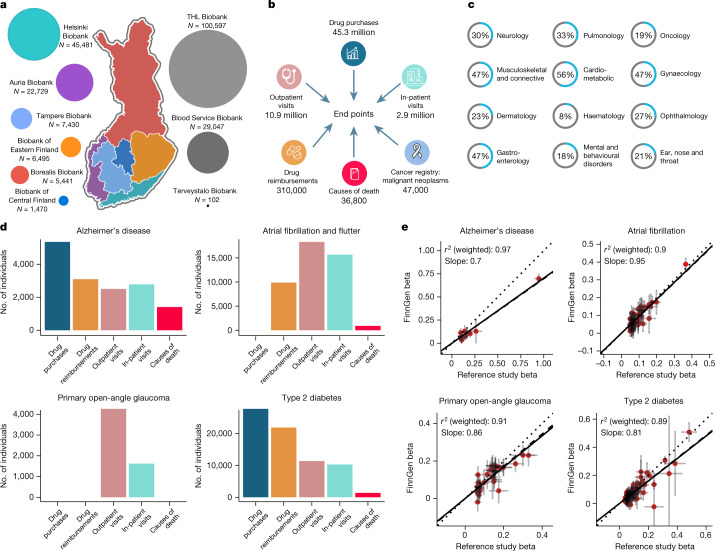
FinnGen sample collection and phenotyping. **a**, Samples collected from different geographical areas. The map of Finland is divided into major administrative areas. Coloured regions represent the areas of the nine biobanks that provide samples to FinnGen. The Finnish Institute for Health and Welfare (THL), the Blood Service and the Terveystalo biobanks are not regional. The circle size represents relative sample sizes. The number of samples given are those used in the analyses after QC. **b**, National registries utilized to construct FinnGen end points. The numbers indicate the number of events in each register at the time of FinnGen release. An individual can have multiple diagnoses and can have events from multiple registers contributing to the end point of the individual. **c**, Sample prevalence of major disease categories in FinnGen. Major diseases for each category were chosen for demonstration purposes (Supplementary Tables 3 and 4). **d**, Examples of registers used for constructing four selected end points. The *y* axis represents individuals with matching register code in each register according to FinnGen end point definitions. Each individual can contribute only once to each register but the same individual can be counted in multiple registers. **e**, Comparison of effect sizes (beta values) in known genome-wide significant loci between four example FinnGen end points and large reference GWAS. The *y* and *x* axes represent FinnGen and reference GWAS beta values respectively. Beta values are aligned to be positive in reference studies. Lines extending from points indicate standard errors of beta values. Regression lines omit intercept and two types of regressions are provided: unweighted and weighted by pooled standard errors from the two studies. Solid line indicates identity line and dotted line and dashed lines indicate unweighted and weighted regression, respectively. Sample sizes used for **e** are given in Supplementary Table 7. Only variants with *P* < 1 × 10^−10^ in reference study were included. A comparison of all 15 diseases is provided in the Supplementary Information. Part **a** adapted with permission from an original biobank map created by BBMRI.fi.

**Extended Data Fig. 1:**
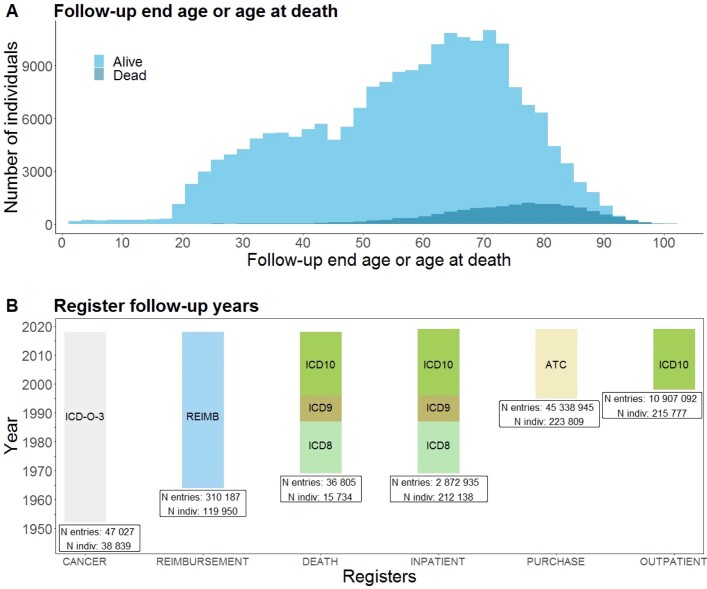
FinnGen Age Distribution and Registers. A) Distribution of the current age (age at the end of the follow-up) and age of death for FinnGen participants B) Follow-up time and main coding used in each register among FinnGen participants in FinnGen release 5. Abbreviations: CANCER = The Finnish Cancer Registry; DEATH = Cause of death register; INPATIENT = HILMO - Care Register for Health Care: Inpatient hospital visits; OUTPATIENT = HILMO - Care Register for Health Care: Specialty outpatient visits and day surgeries; PURCHASE = Drug Purchases: All Prescription drug purchases; REIMBURSEMENT = Drug Reimbursement: entitlements for prescription drug reimbursement for certain chronic diseases.

In Finland, similar to the other Nordic countries, there are nationwide electronic health registers that were originally established primarily for administrative purposes to monitor the usage of health care nationwide and over the lifespan of each Finnish resident. These registers have almost complete coverage of major health-related events such as hospitalizations, prescription drug purchases (not including hospital-administered medications), medical procedures or deaths, with a history of data collection spanning more than 50 years. Phenotypes based on health registers (end points) were created by combining data (mainly using classification codes from the International Classification of Diseases (ICD) and the Anatomical Chemical Therapeutic (ACT)) from one or more nationwide health registers (Extended Data Fig. 1, Supplementary Table 1 and Supplementary Figs. 1–4). For the phenome-wide GWAS, we initially constructed more than 2,800 end points by combining data from different health registers, including hospital discharge registers, prescription medication purchase registers and cancer registers (Fig. 1 and  Supplementary Methods, section 1; see also https://r5.risteys.finngen.fi/).

FinnGen release 5 presented here contains genotype data for 224,737 individuals after quality control (QC). A total of 154,714 individuals were genotyped with a custom Axiom FinnGen1 array. Data on 70,023 additional individuals were derived from legacy collections (Supplementary Table 2) genotyped with non-custom genotyping arrays (QC details provided in  Supplementary Methods, section 3). We developed and utilized a population-specific imputation reference panel of 3,775 high-coverage (25–30 times) whole-genome sequence data for Finnish individuals, containing 16,962,023 single nucleotide polymorphisms, and insertions and deletions (minor allele count of ≥3) ( Supplementary Methods, section 3). The majority (16,387,711) of the variants were confidently imputed (information (INFO) score of >0.6; Supplementary Fig. 5).

## Population structure and relatedness

**Extended Data Fig. 2:**
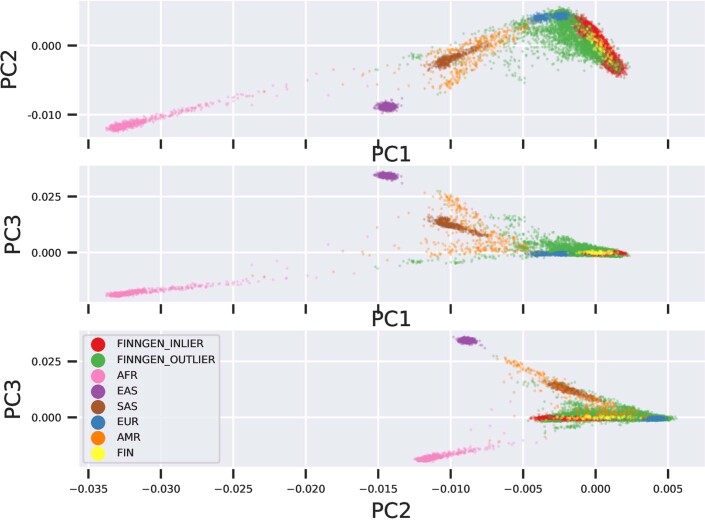
PCA classification of 224,737 FinnGen participants combined with 1000 genomes samples (AFR,AMR,EAST,EUR,FIN,SAS). FinnGen outlier samples were removed as deviating from the bulk of the FinnGen samples.

To study the genetic ancestry data of 224,737 FinnGen participants that passed genotyping QC ( Supplementary Methods, section 3), we combined the FinnGen data with 2,504 phase 3 reference samples from the 1000 Genomes Project^5^ and used principal component analysis (PCA) to identify FinnGen participants who have non-Finnish genetic ancestry. Most participants have broadly Finnish ancestry; 3,676 out of 224,737 (1.63%) outliers were removed (Extended Data Fig. 2 and  Supplementary Methods, section 4). We estimated that 165,448 (73.6%) of FinnGen participants have third-degree or closer relatives, which is higher than the estimated 30.3% in the UK Biobank (UKBB)^6^; this result is partially explained by the family-based legacy cohorts in FinnGen. We removed 5,780 duplicates and monozygotic twins (one from each pair removed randomly) and genetic population outliers (Supplementary Methods, section 4) and built a set of approximately unrelated individuals for which the relation between any pair is third degree or higher. In total, we obtained data for 156,977 independent individuals, which were used to compute the PCA, and data for the 61,980 related individuals were projected onto these principal components (PCs) (Supplementary Methods, section 4, and Supplementary Table 5). The first two PCs captured the well-known east–west and north–south genetic differences in Finland^7^ (Supplementary Fig. 9). Out of the total remaining 218,957 genotyped samples, we had phenotype data for 218,792 individuals (56.5% females (123,579)), which were then used in all analyses.

## GWAS of nationwide health registries

To benchmark our register-based phenotyping and to explore the value of the isolated setting of Finland, we selected 15 diseases with more than 1,000 cases in FinnGen and for which well-powered GWAS data have been published. We evaluated the accuracy of our phenotyping by comparing the genetic correlations and effect sizes with the previous GWAS results (Supplementary Table 6). None of the genetic correlations were significantly lower than 1 (the lowest genetic correlation was 0.89 (standard error = 0.07) in age-related macular degeneration (AMD); Supplementary Table 6). For diseases with a large number of cases in FinnGen, the effect sizes of lead variants in known loci were largely consistent between FinnGen and previously published meta-analyses. This result demonstrates that our register-based phenotyping is comparable to existing disease-specific GWASs (Fig. 1e, Supplementary Information and Supplementary Table 6). The effect sizes varied more in some diseases that have a smaller number of cases in FinnGen (for example, ankylosing spondylitis, *n* = 1462, *r*^2^ = 0.62).

GWAS of these 15 diseases identified 235 loci (that is, regions selected for fine-mapping; Methods) and 275 independent genome-wide significant associations (here onwards, ‘association’ means an independent signal) outside the human leukocyte antigen (HLA) region (GRCh38, chromosome 6: 25–34 Mb). A phenome-wide association study (PheWAS) of FinnGen imputed classical HLA gene alleles has been previously reported^8^. Overall, 44 of the non-HLA associations were driven by low-frequency lead variants (we define ‘low frequency’ as an AF of <5% in non-Finnish, Swedish or Estonian European (NFSEE) individuals in the Genome Aggregation Database (gnomAD; v.2.0.1)^9^) that were more than twice as frequent in Finnish individuals compared with NFSEE individuals. We use NFSEE as a general continental European reference point, excluding individuals from Finland, Sweden and Estonia. As there were large-scale migrations from Finland to Sweden in the twentieth century, many of the chromosomes from sequencing studies of Swedish individuals are of recent Finnish origin. Moreover, the geographically close and linguistically and genetically similar^9^ population of Estonia is likely to share elements of the same ancestral founder effect.

**Fig. 2:**
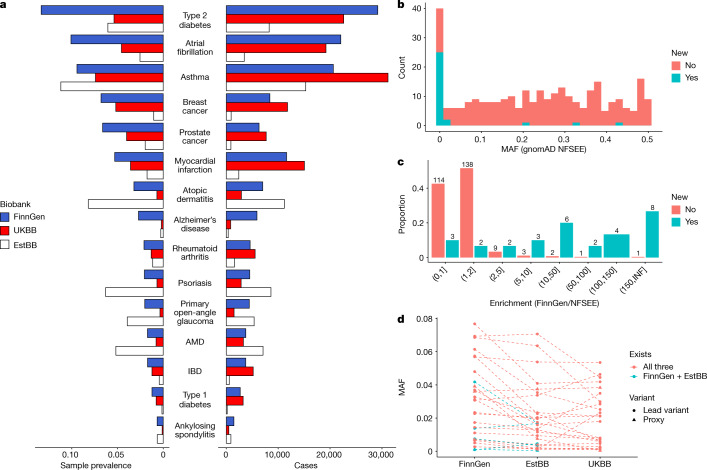
Comparison of previously unknown and known lead variants in loci identified in the 15 studied diseases. **a**, Case prevalence and counts in FinnGen, the EstBB and the UKBB. The phenotypes are sorted on the basis of FinnGen prevalence. **b**, Distribution of minor AFs in known (red) and new (blue) loci in the NFSEE population. **c**, Distribution of AF enrichment between Finland and other Northwestern European populations in gnomAD (excluding Estonia and Sweden). The *x* axis represents enrichment bins. **d**, AFs of 25 replicated genome-wide significant (in FinnGen discovery) new low-frequency (<5% in NFSEE populations) variants in FinnGen, the EstBB and the UKBB. The dotted line indicates the same variants and no line means absence of the variant in other biobanks.

**Extended Data Fig. 3:**
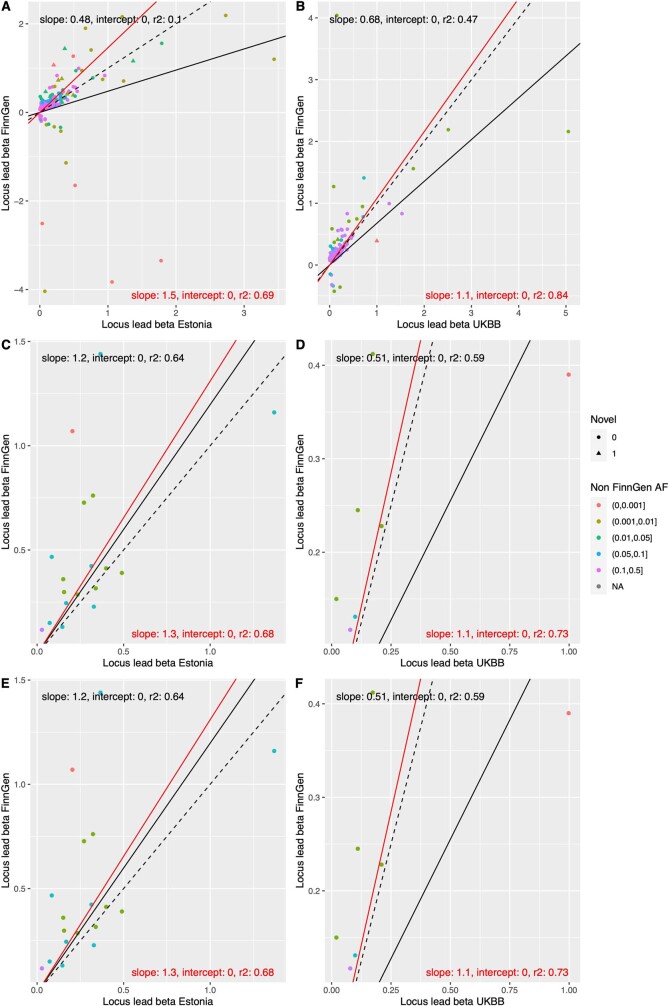
Comparison of effect sizes between biobanks. A,B) Effect size (log(OR), beta) comparison of 275 genome-wide significant lead variants identified in FinnGen among 15 analysed diseases in Estonia and UKBB. The sign of beta is aligned to be positive in Estonia and UKBB. C,D) beta comparison of variants only in known loci. E,F) beta comparison of novel loci. Dashed lines indicates identity line and solid lines are the regression line (red line and text weighted by pooled standard error of betas).

Replication of many such enriched variant associations in the Finnish population is hindered by low AFs or missingness in other European populations. People from Finland are genetically more similar to people from Estonia than other European countries^9^. Therefore we first conducted replication using data from 136,724 individuals from the Estonian Biobank (EstBB) and then extended the analysis to individuals from the UKBB (Methods and see Supplementary Table 7 for definitions of end points and case–control numbers). The effect sizes in genome-wide significant hits in FinnGen were mostly concordant with the EstBB (average inverse variance weighted slope of 1.5 (with FinnGen higher) and *r*^2^ = 0.69) and the UKBB (slope = 1.1, *r*^2^ = 0.84) (Extended Data Fig. 3). FinnGen had a higher case prevalence in the 15 disease diagnoses than in the UKBB, which is probably due to slightly different ascertainment schemes. By contrast, the EstBB had the highest case prevalence in ophthalmic diseases (AMD and glaucoma) and inflammatory skin conditions (atopic dermatitis and psoriasis) (Fig. 2a).

After a meta-analysis of the EstBB and UKBB data, 241 of the 275 associations remained genome-wide significant (Supplementary Table 8). We performed a further meta-analysis of 232 associations that did not meet the genome-wide significance threshold in FinnGen (5 × 10^−8^ < *P* < 1 × 10^−6^), and 57 of those were genome-wide significant after meta-analysis. This meta-analysis resulted in 298 genome-wide significant associations (see also Supplementary Table 8 for results after multiple testing correction for 15 end points).

To determine whether the observed associations have been previously reported, we queried the GWAS Catalog association database (and largest recent relevant GWAS) for genome-wide significant (*P* < 5 × 10^−8^) variants that are in linkage disequilibrium (LD) (*r*^2^ > 0.1 in the FinnGen imputation panel) with observed lead variants in FinnGen. As the lowest AF of the new findings was low (0.15%), in addition to published GWASs, we checked whether credible set variants in these loci have also been previously reported in ClinVar. We observed six known pathogenic or likely pathogenic variants, such as a frameshift variant in *PALB2* (p.Leu531fs; AF of 0.1%, not observed outside Finland in gnomAD; Supplementary Table 8) associated with breast cancer. Thirty out of the 298 associations have not been previously reported in the largest published meta-analysis so far (Supplementary Table 6), in a manual literature search, the GWAS Catalog or in ClinVar (Table 1). As expected, we observed that lead variants in novel loci were mostly of low frequency and enriched in Finland compared with known loci from previous GWASs. Specifically, 27 lead variants had minor allele frequency (MAF) values of <5% in gnomAD NFSEE individuals, and 88% of novel and 11% of known loci (after LD pruning, see below) had gnomAD NFSEE MAF values of <5% (Fisher’s exact test, *P* *=* 4.29 × 10^−17^). In most cases, the AFs of lower frequency variants (MAF < 5% in gnomAD NFSEE population) were the highest in FinnGen followed by the EstBB and lowest in NFSEE individuals in gnomAD (Fig. 2d).

**Table 1:** A total of 30 previously unreported associations identified in a GWAS of 15 selected, previously extensively studied phenotypes

Phenotype	rsID (hg38)^a^	MAF_FinnGen_/ MAF_NFSEE_	Protein change (HGVSp)^b^	Function of variant^c^	Gene^d^	Meta-analysis OR; *P*	FinnGen AF %; OR; *P*	EstBB AF %; OR; *P*	UKBB AF %; OR; *P*
IBD	**rs748670681**	**115.0**		**Intron**	***TNRC18***	**3.2; 2.4** **×** **10**^**−61**^	**3.6; 3.2; 1.1** **×** **10**^**−56**^	**1.3; 3.9; 2.8** **×** **10**^**–**^^**06**^	**NA; NA; NA**
Ankylosing spondylitis	**rs748670681**	**115.0**		**Intron**	***TNRC18***	**3.4; 3.6** **×** **10**^**−31**^	**3.6; 4.2; 1.8** **×** **10**^**−34**^	**1.3; 1.4; 0.11**	**NA; NA; NA**
Type 2 diabetes	**rs45551238**	**9.6**		**5′** **UTR**	***ATP5E***	**0.8; 6.6** **×** **10**^**−24**^	**5.0; 0.8; 2.2** **×** **10**^**−19**^	**1.1; 0.7; 0.001**	**0.7; 0.8; 0.001**
Primary open-angle glaucoma^e^	**rs377027713 (rs147660927, PIP: 0.293)**	**87.4**	**p.Arg220Cys**	**Upstream gene (missense)**	***TARDBP*** **(*ANGPTL7*)**	**0.7; 2.6** **×** **10**^**−14**^	**4.3; 0.6; 1.5** **×** **10**^**−12**^	**1.1; 0.7; 0.003**	**NA; NA; NA**
Type 2 diabetes	**Chromosome** **23: 56173773:A:C**	**3.6**		**Intergenic**		**1.1; 3.2** **×** **10**^**−13**^	**4.8; 1.1; 2.2** **×** **10**^**−10**^	**1.8; 1.2; 0.016**	**1.4; 1.1; 0.005**
Atrial fibrillation	**rs190065070 (rs199600574,** **PIP:0.051)**	**16.6**	**p.Arg1845Trp**	**Intergenic (missense)**	**(*MYH14*)**	**1.4; 2.3** **×** **10**^**−12**^	**2.1; 1.4; 1.9** **×** **10**^**−12**^	**0.6; 1.2; 0.46**	**NA; NA; NA**
Asthma	**rs74630264 (PIP: 0.232)**	**13.6**	**p.Ala82Thr**	**Regulatory region (missense)**	**(*IL4R*)**	**0.9; 1.1** **×** **10**^**−11**^	**8.2; 0.9; 2.5** **×** **10**^**−12**^	**2.9; 0.9; 0.061**	**0.7; 1; 0.72**
Atrial fibrillation	**rs147972626 (PIP: 0.69)**	**2.7**	**p.Arg242Trp**	**Missense**	***RPL3L*** **(*RPL3L*)**	**1.4; 1.1** **×** **10**^**−11**^	**1.3; 1.5; 8.2** **×** **10**^**−11**^	**0.64; 1.5; 0.033**	**0.6; 1.2; 0.017**
Psoriasis	**rs138009430 (rs144651842,** **PIP: 0.211)**	**136.0**	**p.Ala82Thr**	**Regulatory region (missense)**	***FLJ21408*** **(*IL4R*)**	**1.2; 1.9** **×** **10**^**−11**^	**7.9; 1.3; 3.5** **×** **10**^**−9**^	**2.8; 1.2; 0.001**	**0.7; 1.1; 0.51**
Myocardial infarction	**rs534125149 (PIP: 0.232)**	**INF**^**f**^	**p.Asn239dup**	**Inframe insertion**	***MFGE8***	**0.7; 3.8** **×** **10**^**−11**^	**2.9; 0.7; 1.1** **×** **10**^**−10**^	**0.6; 0.7; 0.14**	**NA; NA; NA**
Atrial fibrillation	**rs201864074 (PIP: 0.536)**	**23.1**	**p.Arg4Gln**	**Missense**	***RPL3L***	**1.5; 9.2** **×** **10**^**−11**^	**1.2; 1.5; 1.4** **×** **10**^**−8**^	**0.27; 1.6; 0.1**	**0.04; 2.7; 0.001**
Psoriasis	**rs748670681**	**115.0**		**Intron**	***TNRC1***	**1.4; 1.2** **×** **10**^**−10**^	**3.6; 1.6; 1.2** **×** **10**^**−13**^	**1.3; 1.1; 0.27**	**NA; NA; NA**
Breast cancer	**rs1457477682**	**0.9**		**Intergenic**		**1.1; 1.6** **×** **10**^**−10**^	**32; 1.1; 1.6** **×** **10**^**−10**^	**NA; NA; NA**	**NA; NA; NA**
Type 2 diabetes	**Chromosome** **23: 48591031:T:C**	**1.5**		**Intron**	***WDR13***	**0.9; 2.3** **×** **10**^**−10**^	**2.7; 0.9; 8.6** **×** **10**^**−7**^	**3.0; 0.9; 0.007**	**2.4; 0.9; 0.002**
Type 2 diabetes	**rs190116876**	**57.7**		**Intron**	***CTNNA3***	**1.3; 2.9** **×** **10**^**−10**^	**2.0; 1.4; 3.1** **×** **10**^**−10**^	**0.35; 1.2; 0.53**	**NA; NA; NA**
Type 2 diabetes	**rs540205414**	**35.9**		**Upstream gene**	***SCT***	**1.3; 3.1** **×** **10**^**−10**^	**1.4; 1.3; 2.1** **×** **10**^**−9**^	**0.74; 1.3; 0.048**	**NA; NA; NA**
Type 2 diabetes	**rs1458770448(rs762966411, PIP: 0.141)**	**INF**^**f**^	**p.His293LeufsTer7**	**Intergenic (frameshift)**	**(*RFX6*)**	**3.1; 5.2** **×** **10**^**−10**^	**0.1; 3.1; 5.2** **×** **10**^**−10**^	**NA; NA; NA**	**NA; NA; NA**
Atopic dermatitis	**rs2227472**	**0.9**		**Upstream gene**	***IL22***	**1.1; 5.7** **×** **10**^**−10**^	**55.8; 1.1; 1.8** **×** **10**^**−10**^	**66.1; 0.66; 1; 0.07**	**59.3; 1.1; 0.004**
Type 2 diabetes	rs10835932	0.9		Intergenic		1.1; 7.7 × 10^−9^	18.4; 1.1; 7.2 × 10^−7^	18.7; 1.1; 0.023	20.2; 1; 0.009
Atrial fibrillation	rs755287827 (rs766868752, PIP: 0.131)	9.4	**c.105+1G>T**	Intron (splice donor)	*USP54* (*SYNPO2L*)	2.7; 9.6 × 10^−9^	0.14; 2.9; 3.2 × 10^−9^	0.057; 1.2; 0.71	NA; NA; NA
AMD	rs139779213 (PIP: 0.467)	INF^f^		3′ UTR	*CFI*	2.1; 9.9 × 10^−9^	1.1; 2.0; 1.8 × 10^−7^	0.05; 6.8; 0.002	NA; NA; NA
Breast cancer	rs1171552087	6.2		Intron	*CNTNAP2*	33.1; 1.1 × 10^−8^	0.04; 33.1; 1.1 × 10^−8^	NA; NA; NA	NA; NA; NA
Prostate cancer	rs1301285839	INF^f^		Downstream gene	*SNORA40*	7.1; 1.2 × 10^−8^	0.1; 7.1; 1.2 × 10^−8^	NA; NA; NA	NA; NA; NA
Atopic dermatitis	rs950951813 (rs201208667, PIP: 0.191)	INF^f^	p.Cys379Tyr	3′ UTR (missense)	*SERPINB8* (*SERPINB7*)	1.6; 1.4 × 10^−8^	0.6; 2.1; 5.6 × 10^−9^	0.4; 1.3; 0.021	NA; NA; NA
Type 2 diabetes	rs193302380	13.9		Intron	*SPATS2*	1.1; 1.8 × 10^−8^	6.1; 1.1; 1.7 × 10^−7^	4.2; 1.1; 0.028	0.2; 1; 0.91
Asthma	rs552196550	INF^f^		Intron	*DYNC1I1*	2.0; 2.3 × 10^−8^	0.3; 2.0; 2.3 × 10^−8^	NA; NA; NA	NA; NA; NA
Prostate cancer	rs954957419 (rs965427251, PIP: 0.44)	0.2	p.Ala139_Leu148del	Intron (inframe deletion)	*TTLL1* (*BIK*)	3.5; 2.5 × 10^−8^	0.3; 3.5; 5.4 × 10^−8^	0.09; 3; 0.21	NA; NA; NA
Seropositive rheumatoid arthritis	rs555210673	INF^f^		Intron	*SFRP4*	1.5; 2.7 × 10^−8^	2.3l 1.5; 7.4 × 10^−7^	0.4; 2.7; 0.002	NA; NA; NA
Primary open-angle glaucoma	rs10658374	1.5		Upstream gene	*PAM*	135.6; 2.7 × 10^−8^	0.03; 135.6; 2.7 × 10^−8^	NA; NA; NA	NA; NA; NA
Atopic dermatitis	rs775241954	INF^f^		Intron	*NOTCH2*	1.9; 3.8 × 10^−8^	0.6; 2.1; 2.7 × 10^−8^	0.2; 1.4; 0.16	NA; NA; NA

Table is ordered by meta-analysis *P* values in descending order of significance. All reported variants were mapped to GRCh38. Rows that are in bold are variants surpassing Bonferroni multiple testing correction for 15 end points (*P* < 3.3 × 10^–9^). NA, not applicable; UTR, untranslated region.

^a^The coding variant rsID in PIP is given in parentheses if a coding variant was observed in the credible set (omitted if the reported lead variant was a coding variant).

^b^HGVS notation protein coding change is provided if either the lead variant was coding or coding credible was observed in the credible set (if either one exists).

^c^Coding variant consequence is given in parentheses in cases in which the lead variant was not a coding variant and a coding variant was observed in the credible set.

^d^Gene corresponding to the variant function. In cases in which a lead variant was not a coding variant, but there was a coding variant in the credible set, the credible set coding variant gene is given in parentheses.

^e^We have previously published the *ANGPTL7* variant association with glaucoma^35^.

^f^Denotes values of infinity (INF) resulting from MAF_NFSEE_being 0.00.

Next we performed statistical fine-mapping (Methods) on all 298 genome-wide significant associations (each association is independent; that is, 298 credible sets). Coding variants (missense, frameshift, canonical splice site, stop gained, stop lost or inframe deletion) with posterior inclusion probability (PIP) values of ≥0.05 were observed in 44 (18.7%) out of the 95% credible sets (17 coding variants had PIP > 0.5). Here onwards, we report coding variants with PIP > 0.05 as putatively causal. We recognize that there may be occasions in which assignment of the causal variant to a coding variant is incorrect (see our accompanying paper^10^ for discussions on fine-mapping calibration and replicability). In addition to identifying putative causal coding variants, we sought to identify potential gene expression regulatory mechanisms by colocalizing credible sets with fine-mapped expression quantitative trait locus (eQTL) datasets from the eQTL Catalogue (Methods).

We then wanted to describe the AF spectrum and putative mechanisms of action of risk variants. To do so, we LD pruned the 298 genome-wide significant associations and prioritized the most significant phenotype among the same hits to represent a single putative causal variant (LD *r*^2^ value between lead variants of <0.2). This process resulted in 281 previously unknown associations (27 new).

Most of the 281 previously unknown associations were common variant associations. However, 53 of these had a lead variant frequency of less than 5% in NFSEE individuals, and 38 of them were enriched by more than two times in the Finnish population compared with the NFSEE population. We observed a coding variant more often in the credible sets of associations that were enriched by more than twofold (19 out of 38; 50%) than in non-enriched associations (6 out of 15; 40%) at lower frequencies (MAF < 5%).

Following the discovery of 27 new associations, we sought to determine potential mechanisms of action through the identification of coding variants in their credible sets and potential regulatory effects by colocalization with eQTL associations from the eQTL Catalogue. We identified putative causal coding variants in 9 out of 27 loci and eQTL colocalization in 4 out of 27 loci. In two out of the four eQTL loci, we observed a coding variant in credible sets (*IL4R* and *MYH14*; the eQTLs point to different genes than the coding variants). The two remaining eQTL colocalizations were breast cancer loci colocalizing with *H2BP2* eQTL in lung tissue and type 2 diabetes colocalizing with *PRRG4* in lipopolysaccharide-stimulated monocytes. The disease relevance of these eQTLs is currently not evident.

No credible coding variants or eQTLs were identified in 16 out of 27 loci (Supplementary Table 8). The fraction of associations in which we observed eQTLs was small (14.8%). Most of the new associations were driven by variants with low AFs in NFSEE populations (Table 1 and Fig. 2b,d). The low fraction of observed eQTL colocalizations is probably explained by the low AF of 25 out of the 27 of the variants in available eQTL studies (such as GTEx), for which the majority of the samples do not have Finnish or Estonian ancestry.

We next aimed to explore the benefits of the FinnGen dataset in GWAS discovery. We extrapolated observed meta-analysis results in FinnGen, the UKBB and the EstBB to match the sample size of the UKBB in 14 demonstration diseases (excluding Alzheimer’s disease;  Supplementary Methods). The distribution of extrapolated *P* values was shifted towards greater significance in FinnGen compared with those of the UKBB and the EstBB in a matched total sample size scenario for the 14 demonstration diseases ( Supplementary Methods and Supplementary Fig. 11). Moreover, frequency enrichment was a major driver in the gain of power in low-frequency variants (Supplementary Fig. 12). In individual end points with similar sample prevalence in FinnGen and the UKBB, similar for inflammatory bowel disease (IBD), the greatest gain in power was in variants in which the AFs are <0.5% in the UKBB (see Supplementary Fig. 13 for a comparison for each end point and biobank).

The identification of a new signal for IBD mapping to a single variant in an intron of *TNRC18* highlights the value of FinnGen for discovery, even when the case sample size is below that of existing meta-analyses. This variant has a strong risk-increasing effect (AF = 3.6%, odds ratio (OR) = 3.2, *P* = 2.4 × 10^−61^), which eclipses the significance of signals at *IL23R*, *NOD2* and the major histocompatibility complex. The variant is enriched by 114-fold in the Finnish population compared with the NFSEE population, in whom the AF is too low (0.04%) to have been identified in previous GWASs (this FinnGen association was also reported in ref. ^11^). We were, however, able to replicate this association in the EstBB (AF = 1.3%, OR = 3.9, *P* = 2.8 × 10^−6^) owing to the relatively higher frequency in the genetically related Estonian population. This variant was also associated with risk for multiple other inflammatory conditions evaluated in FinnGen, including interstitial lung disease (OR = 1.43, *P* = 6.3 × 10^−26^), ankylosing spondylitis (OR = 4.2, *P* = 1.8 × 10^−34^), iridocyclitis (OR = 2.3, *P* = 1.2 × 10^−27^) and psoriasis (OR = 1.6, *P* = 1.1 × 10^−13^). However, the same allele appears to be protective for an end point that combines multiple autoimmune diseases (https://r5.risteys.finngen.fi/phenocode/AUTOIMMUNE) (OR = 0.84, *P* = 6.2 × 10^−12^; for example, type 1 diabetes (OR = 0.64, *P* = 2.7 × 10^−7^) and hypothyroidism (OR = 0.85, *P* = 7.8 × 10^−7^).

The highest number (eight loci) of new and enriched low-frequency associations were identified in type 2 diabetes, which is probably due to the large number of patients with type 2 diabetes in FinnGen release 5 (29,193). Other noteworthy observations from this set of 30 findings for 15 well-studied diseases are described in Supplementary Note 1.

### Coding variant associations

Motivated by the identification of high-effect coding variant associations within the selected 15 diseases, we performed a PheWAS followed by fine-mapping to identify putative causal coding variants enriched in the Finnish population.

In a GWAS of 1,932 distinct end points and 16,387,711 variants (Supplementary Table 4; case overlap < 50% and *n* cases > 80), we identified 2,733 independent associations in 2,496 loci across 807 end points (Supplementary Table 9) at a genome-wide significance threshold (*P* < 5 × 10^−8^). Moreover, 893 signals in 771 loci across 247 end points at PWS thresholds (*P* < 2.6 × 10^−11^) were identified. The HLA region was excluded here, and a PheWAS of imputed classical HLA gene alleles in FinnGen is reported in ref. ^8^.

Using statistical fine-mapping, we observed a coding variant (missense, frameshift, canonical splice site, stop gained, stop lost or inframe deletion; PIP > 0.05) in 369 associations (13.5% of all associations) spanning 202 end points. Full results with all 2,803 end points (including end points with a case overlap of >50% that are excluded here) are publicly available from a customized browser based on the PheWeb code base (https://r5.finngen.fi) and as summary statistic files (https://www.finngen.fi/en/access_results).

To put the frequency spectrum and putative mechanisms of action in an interpretable context, we chose a single most-significant association per signal by LD-based merging (*r*^2 ^> 0.3 lead variants merged), which resulted in 1,838 unique associations in 681 end points (Supplementary Table 10). Overall, 493 of the associations in 112 end points were PWS (*P* < 2.6 × 10^−11^). Although most of the 493 PWS unique associations were driven by common variants, 143 and 97 had a lead variant frequency of <5% and <1%, respectively, in gnomAD NFSEE populations. We observed that 82 (57.3%) of the 143 low-frequency (MAF < 5%) lead variants were enriched by more than twofold in Finland compared with NFSEE populations. To estimate the number of putative new associations, we searched for known significant associations using the Open Targets API platform (GWAS Catalogue and the UKBB) and ClinVar for each of the 1,838 associations. Among these, 864 (47%) were not associated with any phenotype in those databases (75 out of 493 (15%) of the stringent *P* < 2.6 × 10^−11^ associations). The fraction of previously unreported associations among genome-wide significant (702 out of 841 (84%)) and stringent (69 out of 143 (48%)) associations were notably higher among low-frequency variants (MAF < 5% in NFSEE individuals).

**Fig. 3:**
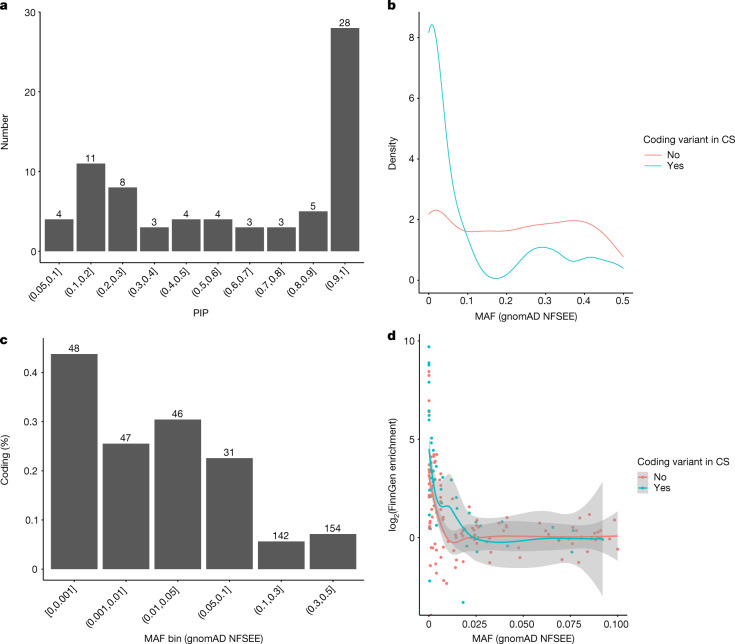
Characteristics of unique associations in end points identified in FinnGen. Characteristics of 493 (73 with coding variants in the credible set) specific associations in 112 (42 end points with coding variants in the credible set) end points identified in FinnGen release 5. Note that 25 of the associations with a coding variant with PIP < 0.05 in credible sets were removed from plots as ‘uncertain to contain coding variant’. **a**, Distribution of fine-mapping PIP values of the 73 coding variants. **b**, AF spectrum in associations with and without coding variants in credible sets (CS). **c**, Proportion of coding variants identified in different AFs (in NFSEE individuals in gnomAD). The numbers above the bars indicate the number of associations within a bin, the *y* axis indicates the proportion of associations with coding variants in their credible sets. **d**, Enrichment in Finland as a function of AF in the gnomAD NFSEE population (enrichment value for variants with AF values of 0 in NFEE individuals in gnomAD was set to maximum observed enrichment value of log_2_(166) = 7.38). The smoothed regression lines of local average enrichment are estimated by local polynomial fitting (loess) and the shaded areas represent 95% confidence intervals of the model fit.

**Extended Data Fig. 4:**
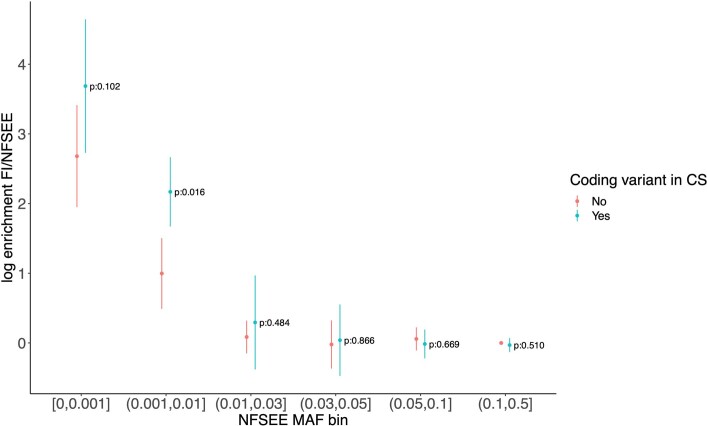
Enrichment of 493 unique phenome-wide significant associations binned by NFSEE MAF and split by whether 95% credible sets contain a coding variant. The p-values of the test of difference in average enrichment are shown on the right side of each MAF bin. Lines indicate 95% confidence interval of the mean enrichment. Number of coding/non-coding variants in each bin : 21/27, 12/35, 11/22, 3/10, 7/24 and 19/277 given in the same order as in the figure x-axis.

After statistical fine-mapping of the 493 unique PWS associations, we identified a coding variant (PIP > 0.05) in 73 (14.8%) of the credible sets associated with 42 end points (Supplementary Table 10). Most (43) of the fine-mapped coding variants had PIP values of >0.5 and 28 had PIP values of >0.9 (Fig. 3a). The highest proportion and the majority (54 out of 73) of associated coding variants had NFSEE MAF < 10% (Fig. 3b,c). The coding variant associations were more enriched in Finland than noncoding associations in associations driven by variants with AFs of <5% in NFSEE people (Fig. 3d; Wilcoxon rank sum test *P* = 3.6 × 10^−3^). For example, we observed a coding variant in 42% (34 out of 89) of the associations with a lead variant that was enriched by more than two times in Finland compared with NFSEE people among low-frequency associations (NFSEE MAF < 5%). By contrast, the proportion of coding variants was lower at 21.7% (13 out of 60) in non-enriched associations (see Extended Data Fig. 4 for enrichment in various NFSEE MAF bins). The higher proportion of coding variants in those that were enriched by more than two times persisted when the PIP threshold was increased to 0.2 (enriched, 30 out of 77 (35.8%); non-enriched, 11 out of 58 (18.9%)).

The fine-mapping properties and replicability of 67 FinnGen traits across diverse biobanks (FinnGen, Biobank Japan and the UKBB) are explored in detail in another manuscript^10^, and functional variant associations in the UKBB and FinnGen are described in ref. ^12^.

We next wanted to quantify the benefits of population isolates such as Finland in GWAS discovery. To this end, we assessed whether lower frequency (MAF < 5% in NFSEE people) variants enriched in the Finnish population were more likely to be associated with a phenotype than would be expected by chance. We randomly sampled 1,000,000 times the number of genome-wide significant variants observed (143) from a set of frequency-matched variants (MAF NFSEE < 5%) that were not associated with any end point (*P* > 0.001). None of the 1 million random draws had a higher proportion of variants enriched by more than twofold in the Finnish population than was observed in the significant associations (57.3% observed versus 33% expected; *P* = 1.0 × 10^−16^).

### Known pathogenic variant associations

Among the genome-wide significant coding variant associations, we identified 13 variant associations (AF range of 0.04–2%) classified as pathogenic or likely pathogenic in ClinVar (Supplementary Table 10). Nine out of the 13 variants were enriched by more than 20-fold in Finland compared with NFSEE populations. Some of these variants have previously been primarily considered recessive. Here, however, we observed that some were a risk variant in the heterozygous state. An example is a rare frameshift variant at *NPHS1* associated with nephrotic syndrome, including the congenital form (ICD-10: N04,p.Leu41fs; AF FinnGen = 0.9%; gnomAD NFSEE = 0.009%; OR = 185, *P* = 4.3 × 10^−27^). Congenital nephrotic syndrome in Finnish individuals is a recessively inherited rare disease, and is in the Finnish Disease Heritage database^4^. The pathogenic variant associations listed in ClinVar include a missense variant in *XPA* (xeroderma pigmentosum) associated with non-melanoma neoplasm of skin (‘other malignant neoplasm of skin’) (p.Arg228Ter; AF FinnGen = 0.02%, gnomAD NFSEE = 0%; OR = 4.4, *P* = 8.3 × 10^−18^), and the abovementioned frameshift variant in *PALB2* associated with breast cancer (p.Leu531fs, ‘malignant neoplasm of breast’; p.Ala82Pro; AF FinnGen = 0.2%, gnomAD NFSEE = 0%; OR = 28.8, *P* = 3.7 × 10^−33^). Furthermore, a known pathogenic recessively acting missense variant in *CERKL* was associated with hereditary retinal dystrophy (p.Cys125Trp; AF FinnGen = 0.6%, gnomAD NFSEE = 0%; OR = 98,716, *P* = 5.15 × 10^−25^). This association is, however, driven by compound heterozygotes, as previously detailed^13^. These associations demonstrate that imputation using a population-specific genotyping array and an imputation panel combined with national-registry-based phenotyping in the isolated Finnish population can successfully identify associations and fine-map causal variants even in rare variants and phenotypes. An extended study of ClinVar variants and variants with specific biallelic Mendelian effects in FinnGen is provided in a companion paper^13^.

### Associations in known disease genes

In the remaining 135 genome-wide significant coding variant associations not reported as pathogenic in ClinVar, 77 had NFSEE MAF values of <5%. Of the 77 variants, 54 were more than 5 times more common in Finland than in NFSEE populations, and 19 had not been previously observed in NFSEE people (Supplementary Table 2). Nine out of the 19 variants are in a gene in which other variants are pathogenic for various traits, 3 of which are for the same or related traits. These FinnGen associations include the following variants: a *RFX6* frameshift variant associated with type 2 diabetes (p.His293LeufsTer7; AF = 0.15%, OR = 3.7, *P* = 1.2 × 10^−10^; ClinVar, ‘monogenic diabetes and others’); a *TERT* missense variant (AF = 0.15%, OR = 1,032, *P* = 6.5 × 10^−21^) associated with idiopathic pulmonary fibrosis (ClinVar, ‘idiopathic pulmonary fibrosis’); a missense in *MYH14* associated with sensorineural hearing loss (p.Ala1156Ser; AF = 0.04%, OR = 19.9, *P* = 1 × 10^−15^; ClinVar, ‘non-syndromic hearing loss’ and others); and a stop gained variant in *TG* associated with autoimmune hypothyroidism (p.Gln655Ter; AF = 0.1%, OR = 3.2, *P* = 3.9 × 10^−11^). These variants in *RFX6, TERT* and *TG* have been previously observed in Finnish and Nordic cohorts^14–16^, but had uncertain significance (single carrier in *TG*) or conflicting interpretation (*TERT*) in ClinVar. Pathogenic variants in *RFX6* cause Mitchell–Riley syndrome with recessive inheritance (characterized by neonatal diabetes). However, heterozygote enrichment of *RFX6*-truncating variants have been observed in maturity-onset diabetes of the young^14^, for which the same variant observed here was identified in a replication in Finnish data. RFX6 is a regulator of transcription factors involved in beta-cell maturation and has a specific role in releasing gastric inhibitory peptide (GIP) and GLP1 in response to meals. Our results propose that around 1:700 individuals in Finland carry a frameshift variant that has been previously shown to reduce incretin levels and to lead to isolated diabetes^14^. It is tempting to speculate that early administration of GLP1 analogues would benefit carriers of this diabetes-associated variant.

### New disease associations

Among the previously undescribed genome-wide significant coding variant associations without previous associations in Open Targets (GWAS Catalog and the UKBB) or ClinVar, we observed 29 that had NFSEE MAF values of <5% and were 2 times more frequent in Finland, 9 of which had no copies in NFSEE populations (Supplementary Table 11). We summarize selected new discoveries and biological knowledge gained in Supplementary Table 12. A missense variant not observed outside Finland (p.Val70Phe; AF = 0.2%, OR = 3.0, *P* = 2.1 × 10^−9^) in *PLTP* was associated with coronary revascularization (*n* = 12,271 coronary angioplasty or bypass grafting). *PLTP* is a lipid-transfer protein in human plasma that transfers phospholipids from triglyceride-rich lipoproteins to high-density lipoprotein, and its activity is associated with atherogenesis in humans and mice^17^. Noncoding variations near *PLTP* independent of p.Val70Phe are associated with lipid levels (high-density lipoprotein and triglycerides)^18^ and coronary artery disease^19^. The identification of a coding variant in this gene provides support for *PLTP* as the causal gene for symptomatic atherosclerosis in this locus. Other variants associated with coronary artery disease included a missense variant (p.Gly567Arg; AF = 0.9%, OR = 2.0, *P* = 5.2 × 10^−12^) in *HHIPL1*, which was associated with coronary revascularization (*n* = 12,271), and a splice acceptor variant (c.7325-2A>G; AF = 0.7%, OR = 2.5, *P* = 2.9 × 10^−08^) in *NBEAL1*, which was associated with coronary artery bypass grafting (*n* = 5,779). Both genes are susceptibility loci for coronary artery disease^19^ and have been suggested as causal, although for *NBEAL1* the evidence is inconsistent^20^. *HHIPL1* encodes a secreted sonic hedgehog regulator that modulates atherosclerosis-relevant smooth muscle cell phenotypes and promotes atherosclerosis in mice^21^. *NBEAL1* regulates cholesterol metabolism by modulating low-density lipoprotein (LDL) receptor expression, and genetic variants in *NBEAL1* are associated with decreased expression of *NBEAL1* in arteries^22^. Our results strengthen the evidence that both these genes are causal in the loci.

A missense variant in *LAG3* (p.Pro67Thr; AF = 0.08%, gnomAD NFSEE = 0%) was associated with autoimmune hypothyroidism (*n* = 22,997, OR = 3.2, *P* = 4.6 × 10^–8^, lead variant *P* = 4.57 × 10^–8^). *LAG3* encodes an immune checkpoint protein that is involved in inhibitory signalling of immune response, especially in T cells^23^. LAG3 has been a target of active immune checkpoint inhibitor cancer immunotherapy development. One such immunotherapy was recently approved by the US Food and Drug Administration as a combination treatment for unresectable or metastatic melanoma^24^. Immune checkpoint inhibition therapies aim to enhance immune responses against tumour cells. Excessive immune responses, however, can exert deleterious effects on healthy tissue and lead to autoimmune disease. A common side effect of immune checkpoint inhibitors, including those that target LAG3, is hypothyroidism. The p.Pro67Thr variant could be acting as an inhibitor of LAG3 immunoregulatory activity, which in turn leads to susceptibility to hypothyroidism. In a PheWAS of p.Pro67Thr, we observed a nominally increased risk for other immune-related conditions (for example, psoriatic arthropathies (M13_PSORIARTH_ICD10) *n* = 1,455, OR = 7.8, *P* = 3.3 × 10^−3^; urticaria and erythema (L12_URTICARIAERYTHEMA), *n* = 6,328, OR = 3.7, *P* = 2.7 × 10^−4^; and streptococcal septicaemia (AB1_STREPTO_SEPSIS), *n* = 1,090, OR = 15, *P* = 2.2 × 10^−3^), but we did not observe protective effects with any cancers. It should be noted, however, that owing to the rarity of the variant, the data were not sufficiently powered to detect more subtle effects.

We found a missense variant (p.Tyr212Phe, rs35937944) in *COLGALT2* that was enriched by >20-fold in the Finnish population. This variant was associated with a reduced risk for arthrosis (OR = 0.79, *P* = 2.57 × 10^−10^), coxarthrosis (OR = 0.68, *P* = 1.34 × 10^−19^) and gonarthrosis (OR = 0.80, *P* = 7.5 × 10^−7^). A noncoding variant near *COLGALT2* has recently been described as a GWAS locus for osteoarthritis^25^. *COLGALT2* encodes the procollagen galactosyltransferase 2, which initiates post-translational modification of collagens by transferring β-galactose to hydroxylysine residues, an important step to ensure structure and function of bone and connective tissue. Modulating COLGALT2 enzymatic activity with drugs could be a potential strategy to reduce arthritis risk.

CD63 is a cell surface protein involved in basophil activation and mast cell degranulation. We identified a missense variant in *CD63* (rs148781286) that was enriched by >42-fold in the Finnish population. This variant was associated with childhood asthma (OR = 3.5, *P* = 3.37 × 10^–9^). In a combined analysis with data from the EstBB and the UKBB, this variant was also associated with atopic dermatitis^26^. Mediators secreted by basophils and mast cells correlate with asthma severity in the clinic, and a CD63-based basophil activation test has been reported to predict asthma outcome in young children with wheezing episodes^27^. The observation of a putative causal relationship between genetic variations in *CD36*, basophil activation and childhood asthma risk and severity may point to a new intervention point for targeted asthma therapies.

A missense variant in *TUBA1*C (p.Ala331Val; AF = 0.2%, OR = 35.2, *P* = 1.4 × 10^−10^) was associated with sudden idiopathic hearing loss (*n* = 1,491). No relevant phenotype has previously been reported for variants in *TUBA1C*. *TUBA1C* encodes an α-tubulin isotype. The precise roles of α-tubulin isotypes are unknown, but mutations in other tubulins can cause various neurodevelopmental disorders^28^. The p.Ala331Val variant was also associated with vestibular neuritis (inflammation of the vestibular nerve; *n* = 1,224, OR = 40.9, *P* = 3.2 × 10^−10^). Pure vestibular neuritis presents acutely with vertigo but not hearing loss, and accurate diagnosis of vertigo in acute settings is challenging and misdiagnosis is possible.

A >30-fold-enriched missense variant, pThr155Met (rs145955907), in *ZAP70* was associated with sarcoidosis (OR = 2.05, *P* = 1.03 × 10^−8^). Previously, homozygote or compound heterozygote mutations in *ZAP70* have been described in cell-mediated combined immunodeficiency caused by abnormal T cell receptor signalling^29^. Associations of heterozygote variants have not been associated with any disease so far. Given its crucial role in cell signalling, the ZAP70 association with sarcoidosis seems in line with its key role in immunity.

A 75-fold-enriched missense variant, p.Ala777Thr (rs199680517), in *PPP1R26* was associated with endometriosis (OR = 1.97, *P* = 3.41 × 10^−8^). PPP1R26 (protein phosphatase 1 regulatory subunit 26) has been associated with tumour formation and has been observed to be upregulated in various malignancies. Cellular GWAS analyses have identified one variant to be associated with carboplatin-induced toxicity^30^. In one study, a copy number variant has been associated with endometriosis, but how this gene contributes to endometriosis susceptibility remains speculative^31^.

We also report several of these coding associations in separate manuscripts. One such new observation is a missense variant (p.Arg20Gln; AF = 3%, gnomAD NFSEE = 0.7%) in *SPDL1* with a pleiotropic association. It is associated with a strongly increased risk of idiopathic pulmonary fibrosis (OR = 3.1, *P* = 1.0 × 10^−15^) but protective with an end point that combines all cancers (OR = 0.82, *P* = 2.1 × 10^−15^)^32^. Other associations between variants and disease described in separate manuscripts include the following: an inframe deletion in *MFGE8* and coronary atherosclerosis (p.Asn239dup; AF = 2.9%, gnomAD NFSEE = 0%, OR = 0.74, *P* = 5.4 × 10^−15^)^33^; a frameshift variant in *MEPE* (p.Lys101IlefsTer26; AF = 0.3%, gnomAD NFSEE = 0.07%, OR = 18.9, *P* = 1.5 × 10^−11^) and otosclerosis^34^; and a missense variant in *ANGPTL7* (p.Arg220Cys; AF = 4.2%, gnomAD NFSEE = 0.06%, OR = 0.7, *P* = 7.2 × 10^−16^) and glaucoma^35^.

### Coding variants associated with drug use

An notable registry available in FinnGen is a prescription medication purchase registry (KELA; Supplementary Table 1), which links all prescription medication purchases for all FinnGen participants since 1995. Using prescription records from this registry, we identified two enriched low-frequency coding variants that were associated with drug purchase of statin medications (three or more purchases per individual) (Supplementary Table 11). A missense variant in *TM6SF2* (p.Leu156Pro, rs187429064) was associated with a decreased likelihood of being prescribed statins (AF = 5.2%, gnomAD NFSEE = 1.2%; OR = 0.86, *P* = 3.8 × 10^−13^) but with an increased likelihood for insulin medication for diabetes (OR = 1.17, *P* = 8.2 × 10^−11^) and type 2 diabetes (OR = 1.15, *P* = 2.6 × 10^−8^). In addition, the same variant showed a strong association with a strongly increased risk of hepatocellular carcinoma (ICD-10 C22 ‘hepatic and bile duct cancer’; OR = 3.7, *P* = 5.9 × 10^−10^). The hepatic and bile duct cancer association did not change after conditioning on statin medication (OR = 3.7, *P* = 7.1 × 10^−10^). Consistent with a decrease in the likelihood of being prescribed statins, *TM6SF2* p.Leu156Pro and another independent (*r*^2^ = 0.003) missense variant (p.Gly167Lys, rs58542926) have previously been associated with decreased LDL and total cholesterol levels^36^. In a mouse model, both p.Gly167Lys and Leu156Pro lead to increased protein turnover and reduced cellular TM6SF2 levels^37^. *TM6SF2* p.Gly167Lys leads to decreases in hepatic large, very LDL particle secretion and increases in intracellular lipid accumulation^38^. These effects probably explain its associations with non-alcoholic fatty liver disease^39^, alcohol-related cirrhosis^40^, hepatocellular carcinoma^41^ and incident type 2 diabetes^42^. Our results provide, in a single PheWAS analysis, strong evidence of a previously unknown p.Leu156Pro variant that has similar consequences of decreasing circulating lipid levels and increasing the risk of diabetes, cirrhosis and liver cancer, as observed for p.Gly167Lys. Such pleiotropy of the variant can be explored in the custom PheWeb browser (http://r5.finngen.fi/variant/19-19269704-A-G).

## Conclusions

In this paper and accompanying publications, we present FinnGen, one of the largest nationwide genetic studies with access to comprehensive electronic health register data of all participants. The final aim of the study is to collect data for 500,000 biobank participants by the end of 2023. The interim releases of FinnGen have already contributed to many new discoveries and insights into human genetic variation and how it affects disease and health^35,43–47^, including contributions to the COVID-19 host genetics initiative^48^ and the global biobank meta-analysis initiative^49^. Summary statistics from each data release will be made publicly available after a 1-year embargo period, and all summary statistics described here are freely available at www.finngen.fi/en/access_results.

An important feature of FinnGen compared with other similar projects, such as the UKBB^6^, is the specific genetic makeup of the Finnish population. In the GWAS of selected, well-studied diseases, we were able to identify several new associations with a fraction of the cases compared with the largest published GWAS. These associations were largely observed with variants that were increased in frequency in the Finnish population bottleneck and would have required prohibitively large sample sizes in older, non-bottlenecked populations (Fig. 2d).

Moreover, in the GWAS of 1,932 end points, we observed that variants in the Finnish population that were enriched by more than twofold were 1.7-times more likely to be associated with a phenotype than would be expected by chance.

Furthermore, we observed that putative coding variant associations were not only of lower AF but also more often enriched in Finland than noncoding variant associations (Fig. 3). This observation is expected, as coding variant associations are more deleterious on average and selection drives the AFs down. However, some of these deleterious alleles survived the bottleneck and increased in frequency, which facilitated the identification of their associations with diseases.

Imputation with a population-specific imputation panel provides high imputation accuracy down to very low AFs (Supplementary Fig. 5), which enabled the identification of associations with low-frequency variants using a GWAS approach instead of direct sequencing. This high imputation accuracy combined with broad population registry-based phenotyping facilitates the identification of very low-frequency variants associated with rare phenotypes, which have largely been missed in the majority of GWASs published so far^50^. We demonstrated this by identifying known ClinVar variant associations with diseases such as congenital nephrotic syndrome or polycystic liver disease, which are both registered in the Finnish Disease Heritage database. Furthermore, we uncovered new low-frequency variant associations with common and rare phenotypes, including clinically challenging but not well genetically studied sudden idiopathic hearing loss or carpal tunnel syndrome. The recently reported^35^ Gln175His variant in *ANGPT7*, which is enriched in the Finnish population and is protective against glaucoma, is also an example of the benefit of the bottleneck effect in the discovery of disease-associated variants.

The university-hospital-based recruitment, together with legacy case cohorts of several diseases, is another feature of FinnGen. This strategy captures cases in many disease areas and distinguishes it from many working-age population cohorts. For example, in the UKBB, in which recruitment was based on postal invitation to individuals aged 40–69 years and living within 40 km (25 miles) of one of the assessment centres^51^, the participants are likely to be healthier than in hospital-based collections. The approach in FinnGen has advantages and disadvantages. For many disease-focused studies, it provides a higher number of cases and a relatively economical way of recruiting a large sample within a feasible time frame. For example, in the 15 common diseases studied in this paper, the sample prevalence in FinnGen was higher than in the UKBB. The difference was the most extreme for Alzheimer’s disease (2.7% in FinnGen compared with 0.2% in UKBB), a disease of old age, and the most similar in asthma (9.4% in FinnGen compared with. 7.4% in the UKBB) (Fig. 2a). FinnGen also has a relatively high sample prevalence of severe mental disorders such as schizophrenia (2.5%, *n* = 5,562) and bipolar disease (2.1%, *n* = 4,501), which are often underrepresented in biobank studies. A key aspect of the recruitment strategy for the Finnish biobank is that legislation enables participants to donate samples with broad consent to medical research in general. This makes recruitment cost-effective, as the same samples and data can be used, after appropriate application steps, for many medical research studies. However, owing to the recruitment strategy, FinnGen is not epidemiologically representative, and some disease prevalence estimates might be over or underrepresented in FinnGen compared with population values (for example, asthma is 10.4% in FinnGen, 7.7 in FinRegistry, and type 2 diabetes is 14.5% in FinnGen, 8.2% in FinRegistry (https://www.finregistry.fi/)). The recruitment strategies for FinnGen are not anticipated to cause significant biases to the GWAS results presented here, but would be an aspect to consider, for example, when studying disease progression or building predictive models. We further explored the benefit of the FinnGen approach and showed that data from FinnGen has greater discovery power than data from the UKBB in a matched sample size scenario for 14 common diseases (Supplementary Fig. 11).

In conclusion, FinnGen as a large-scale biobank resource with specific features of the Nordic healthcare system and population structure provides opportunities for a wide range of genetic discoveries. These include identification of disease-associated coding variants, identification of variant pleiotropy and longitudinal analyses of disease trajectories. Combining results with other large-scale biobank projects can further improve our understanding of the role of genetic variation in health and disease, especially in genetically understudied diseases.

## Methods

### Biobank samples

The FinnGen study (https://www.finngen.fi/en) is an ongoing research project that utilizes samples from a nationwide network of Finnish biobanks and digital healthcare data from national health registers. FinnGen aims to produce genomic data with linkage to health register data of 500,000 biobank participants. Samples in the FinnGen study include legacy samples (prospected number 200,000) from previous research cohorts (often disease-specific) that have been transferred to the Finnish biobanks, and prospective samples (prospected number 300,000) collected by biobanks across Finland. Prospective samples from six regional hospital biobanks represent a wide variety of patients enrolled in specialized health care, samples from a private healthcare biobank enable enrichment of the FinnGen cohort with patients underrepresented in specialized health care, whereas participants recruited through the Blood Service Biobank enrich the cohort with healthier individuals. Samples have not specifically been collected for FinnGen, but the study has incorporated all that have been available in the biobanks (see  Supplementary Methods for details). In the current study, we included samples from 224,737 biobank participants.

### Phenotyping

Registry data on all FinnGen participants were collected and processed from the following different national health registers: hospital and outpatient visits in HILMO, a care register for health care (in-patient and outpatient primary and secondary diagnoses: ICD-8, ICD-9 and ICD-10; operations: NOMESCO Classification of Surgical Procedures and Hospital League surgical procedure codes); AvoHILMO, a register of primary health care (main and secondary diagnosis using ICD-10 and ICPC2 codes, operations and procedures using NOMESCO and national SPAT codes); Cause of Death (immediate, underlying and contributing causes of death on the death certificate with ICD-8, ICD-9 and ICD-10 codes); reimbursed medication entitlements and prescribed medicine purchases (specific Social Insurance Institution of Finland reimbursement codes and ATC codes, respectively); and the Finnish Cancer Registry (using ICD-O-3 codes). Pseudonymized register data were combined with the minimum phenotype dataset from the Finnish biobanks (age, sex, year of sampling, height, weight and smoking status). Clinical end points were constructed from the register codes using the Finnish version of the International Classification of Diseases, 10th revision (ICD-10) diagnosis codes and harmonizing those with definitions from ICD-8 and ICD-9. The Finnish ICD version is mostly identical to the international ICD classification, but has minor modifications. For example, there are additions to certain disease classifications in the fourth and fifth character level to add specificity. When relevant, the information on reimbursed medication and/or prescription medicine purchases and operations augmented the end point data. Cancer end points were constructed on the basis of the Finnish Cancer Registry and Cause of Death data. The definitions of FinnGen disease end points and their respective controls for each release are available at https://www.finngen.fi/en/researchers/clinical-endpoints, and FinnGen end points can also be browsed at https://r5.risteys.finngen.fi/. See  Supplementary Methods, section 1 for further details.

Some of the end points have a high number of overlapping cases. Therefore, to avoid reporting highly repetitive end points, we clustered all end points if there was an overlap of >50% of cases between them and chose the one with the most genome-wide significant hits. On a few occasions, a manual choice was made to select the most representative end point among the correlating end points. After clustering, we had 1,932 end points for the main GWAS analysis.

### Genotyping and QC

Samples were genotyped with Illumina (Illumina) and Affymetrix arrays (Thermo Fisher Scientific). Genotype calls were made with GenCall and zCall algorithms for Illumina and the AxiomGT1 algorithm for Affymetrix data. Chip genotyping data produced with previous chip platforms and reference genome builds were lifted over to build v.38 (GRCh38/hg38) following a previously described protocol^52^. In sample-wise QC, individuals with genetically inferred sex not matching the reported sex in registries, high genotype missingness (>5%) and excess heterozygosity (±4 standard deviations) were removed. In variant-wise QC, variants with high missingness (>2%), low Hardy–Weinberg equilibrium (*P* <1 × 10^–6^) and minor allele count < 3 were removed. Chip-genotyped samples were pre-phased with Eagle v.2.3.5 (https://data.broadinstitute.org/alkesgroup/Eagle/) using default parameters, except the number of conditioning haplotypes was set to 20,000.

### Genotype imputation with a population-specific reference panel

The population-specific Sequencing Initiative Suomi (SISu) v.3 imputation reference panel was developed by using high-coverage (25–30 times) whole-genome sequencing data for 3,775 Finnish individuals. In brief, the variant call set was produced using the GATK HaplotypeCaller algorithm by following GATK best practices for variant calling. Genotype-wise, sample-wise and variant-wise QC was performed using the Hail framework (https://github.com/hail-is/hail) v.0.1, and the resulting high-quality whole-genome sequencing data were phased (Supplementary Methods). Genotype imputation was carried out using the SISu v.3 reference panel with Beagle 4.1 (v.08Jun17.d8b, https://faculty.washington.edu/browning/beagle/b4_1.html) as described in a previous protocol^53^. Post-imputation QC involved non-reference concordance analyses, checking expected conformity of the imputation INFO values distribution, MAF differences between the target dataset and the imputation reference panel, and checking chromosomal continuity of the imputed genotype calls. After these steps, variants with imputation INFO scores of <0.6 or MAF values of <0.0001 were excluded.

### Association analysis and fine-mapping

The mixed-model logistic regression method SAIGE (v.0.35.8.8)^54^ was used for association analysis. We used sex, age, genotyping batch and ten PCs as covariates (see  Supplementary Methods for details). We used SuSiE^55^ for fine-mapping. We fine-mapped all regions with variants that had values of *P* < 1 × 10^−6^ and extended regions 1.5 Mb upstream and downstream from each lead variant. Finally, overlapping regions were merged and subjected to fine-mapping. The major histocompatibility complex region (chromosome 6: 25–36 Mb) was excluded owing to its complex LD structure. We allowed up to ten independent signals per region, and SuSiE reports a 95% credible set for each independent signal. As LD, we used in-sample dosages (that is, cases and controls used for each phenotype) computed with LDStore2. The FinnGen fine-mapping pipeline is available in GitHub (https://github.com/FINNGEN/finemapping-pipeline).

To define independent signals within a locus, we utilized fine-mapping results. For each locus, we report the credible set as an independent hit if it represents a primary strongest signal with lead *P* < 5 × 10^−8^. For secondary hits, we required genome-wide significance and log Bayes factor (BF) > 2. The BF filtering was necessary because SuSiE sometimes reports multiple credible sets for a single strong signal but this is indicated in SuSiE as a low BF (the model does not improve by adding another signal in the region that is not an independent signal).

### Browser development

The https://r5.finngen.fi browser was developed based on the PheWeb^56^ codebase.

### Estimation of expected number of enriched variant associations

We aimed to estimate whether we observed variant associations that were enriched by more than twofold in the Finnish population in the lower frequency range (NFSEE MAF < 5%) than would be expected by chance. To this end, we sampled a subset of variants (NFSEE MAF < 5%) that were not associated with any end point in FinnGen (*P* > 0.001). We drew 1 million random samples of the number of independent hits (143) observed in a GWAS from the set of non-associated variants. To closely follow the observed frequency distribution, we further matched the random samples to contain the same number of variants in each frequency bin ((0,0.001], (0.001,0.005], (0.005,0.01] and then in 0.01 bins up to 0.05). We computed the mean and standard deviations of per cent twofold enriched variants from the random samples and calculated *P* values from the normal distribution using the randomized mean and standard deviation.

### EstBB and UKBB replication

The EstBB is a population-based biobank at the Institute of Genomics, University of Tartu. The current cohort size is 200,000 individuals (aged ≥18 years), reflecting the age, sex and geographical distribution of the adult Estonian population. Overall, 83% of the samples are from Estonian individuals, 14% from Russian people and 3% from other ethnicities. All participants were recruited by general practitioners, physicians in hospitals and during promotional events. After recruitment, all participants completed a questionnaire about their health status, lifestyle and diet. Specifically, the questionnaire included personal data (place of birth, place(s) of living, nationality, among others), genealogical data (family history of medical conditions spanning four generations), educational and occupational history, and lifestyle data (physical activity, dietary habits (food frequency questionnaires), smoking status, alcohol consumption, women’s health and quality of life). The EstBB database is linked with national registries (such as the Cancer Registry and Causes of Death Registry), hospital databases and the database of the national health insurance fund, which holds treatment and procedure service bills. Diseases and health problems are recorded as ICD-10 codes and prescribed medicine according to the ATC classification. These health data are continuously updated through periodical linking to national electronic databases and registries. All participants were genotyped with genome-wide chip arrays and further imputed with a population-specific imputation panel consisting of 2,244 high-coverage (30 times) whole-genome sequence data from individuals and 16,271,975 high-quality variants^57^. Researchers at the EstBB ran an association analysis of the 15 phenotypes (Supplementary Table 8) used in this study in 136,724 individuals. The association analysis was conducted with SAIGE52 mixed models with age, sex and ten PCs used as covariates.

We used the Pan UKBB (https://pan.ukbb.broadinstitute.org/) project European subset association analysis summary statistics in the UKBB replication^58^ (Supplementary Table 7).

As both the EstBB and the UKBB are on human genome build 37, we lifted over the coordinates to build 38 to match FinnGen. Variants were then matched on the basis of chromosome, position, reference and alternative alleles.

Inverse variance weighted meta-analysis was used to perform a meta-analysis on the three cohorts (code available at https://github.com/FINNGEN/META_ANALYSIS).

### Variant annotation

We utilized Variant Effect Predictor (https://www.ensembl.org/info/docs/tools/vep/index.html) for annotating imputation panel variants. For coding variants, we chose a single most-severe consequence and corresponding gene among canonical transcripts. We considered stop gained, frameshift variant, splice donor, splice acceptor, missense variant, start lost, stop lost, inframe insertion and inframe deletion as coding variants. We executed the variant annotation using Hail^59^.

#### Colocalization

We applied colocalization to all fine-mapped regions. As a colocalization approach, we used the probabilistic model for integrating GWAS and eQTL data presented in eCAVIAR^60^. Given the PIP values of each phenotype in a region of interest, we calculated the colocalization posterior probability (CLPP). In contrast to eCAVIAR, we used SuSiE^55^ to estimate the posterior inclusion probabilities.

For a pair of phenotypes, we searched for an intersection of variants between their credible sets CS_*k*_, *k* = 1…*k*, and computed the CLPP as follows:

$${{\rm{CLPP}}}_{k}=\sum _{{\rm{i}}}{\rm{in}}\,{{\rm{CS}}}_{k}\,{\rm{p}}{1}_{{\rm{i}}}\,\times \,{\rm{p}}{2}_{{\rm{i}}},$$

where p1 and p2 are the PIP values from phenotypes 1 and 2, respectively.

We performed colocalization between FinnGen end points, the eQTL Catalogue^61^ and selected 36 continuous end points and 57 biomarkers from the UKBB^10^. eQTL Catalogue and UKBB traits were processed with a functionally equivalent fine-mapping pipeline^10^ to FinnGen and ref. ^61^, and credible sets provided by those studies were used in colocalization.

#### Annotating putatively new associations

For each association lead variants, we used the Open Targets^62^ API platform (https://api.platform.opentargets.org/) to search whether any genome-wide significant hits (*P* < 5 × 10^−8^) have been reported for the variant (or tagging LD variants *r*^2^ > 0.2) in the GWAS Catalog or the UKBB as harmonized by Open Targets (annotated 19 May 2022). We also searched whether the variant was reported as pathogenic or likely pathogenic in ClinVar^63^ (ClinVar release date 7 May 2022).

#### Automatic annotation of known GWAS hits

To identify new hits from the GWAS results, we compared the fine-mapped results against genome-wide significant hits (*P* < 5 × 10^−8^) in the GWAS Catalog association database^64^ and manually curated genome-wide significant hits from large GWASs (Table 1). We checked and reported separately matches in credible set variants and matches with any variants in LD with a lead variant (highest PIP) after fine-mapping. LD lookup variants were chosen using the following criteria: (1) they were less than 1,500 kb away from the lead variant; (2) they had a *P* < 0.01; (3) and their LD squared Pearson’s correlation with the lead variant was higher than a dynamic LD threshold based on the *P* value of the lead variant so that the expected *P* value of the linked variant would be nominally significant (*r*^2^ = 5/inverse chi-squared survival function (*P* value)).

A variant was considered to be already associated if its chromosome and position were identical to the GWAS Catalog association and if its reference and alternative allele matched the strand-aligned and effect-aligned association alleles. Because the GWAS Catalog associations do not have complete allele information, the allele information for associations was retrieved from dbSNP data, human genome build 153, assembly 38. The GWAS Catalog version used was released on 21 April 2021.

### Ethics statement

Participants in FinnGen provided informed consent for biobank research on basis of the Finnish Biobank Act. Alternatively, separate research cohorts, collected before the Finnish Biobank Act came into effect (in September 2013) and the start of FinnGen (August 2017) were collected on the basis of study-specific consent and later transferred to the Finnish biobanks after approval by Fimea, the National Supervisory Authority for Welfare and Health. Recruitment protocols followed the biobank protocols approved by Fimea. The Coordinating Ethics Committee of the Hospital District of Helsinki and Uusimaa (HUS) approved the FinnGen study protocol (number HUS/990/2017).

The FinnGen study is approved by the THL (approval number THL/2031/6.02.00/2017, amendments THL/1101/5.05.00/2017, THL/341/6.02.00/2018, THL/2222/6.02.00/2018, THL/283/6.02.00/2019 and THL/1721/5.05.00/2019), the Digital and Population Data Service Agency (VRK43431/2017-3, VRK/6909/2018-3 and VRK/4415/2019-3), the Social Insurance Institution (KELA) (KELA 58/522/2017, KELA 131/522/2018, KELA 70/522/2019 and KELA 98/522/2019) and Statistics Finland (TK-53-1041-17).

The Biobank Access Decisions for FinnGen samples and data utilized in FinnGen Data Freeze 5 include the following datasets: THL Biobank BB2017_55, BB2017_111, BB2018_19, BB_2018_34, BB_2018_67, BB2018_71, BB2019_7, BB2019_8 and BB2019_26; Finnish Red Cross Blood Service Biobank 7.12.2017; Helsinki Biobank HUS/359/2017; Auria Biobank AB17-5154; Biobank Borealis of Northern Finland_2017_1013; Biobank of Eastern Finland 1186/2018; Finnish Clinical Biobank Tampere MH0004; Central Finland Biobank 1-2017; and Terveystalo Biobank STB 2018001.

### Reporting summary

Further information on research design is available in the Nature Portfolio Reporting Summary linked to this article.

## Online content

Any methods, additional references, Nature Portfolio reporting summaries, source data, extended data, supplementary information, acknowledgements, peer review information; details of author contributions and competing interests; and statements of data and code availability are available at 10.1038/s41586-022-05473-8.

### Supplementary information

Supplementary Information Comparison of effects size in known genome-wide significant loci between FinnGen and large published reference GWASs (Table 1). The *y* and *x* axes represent FinnGen and reference GWAS effect sizes, respectively. Beta values are aligned to be positive in reference studies. Lines extending from points indicate standard errors in respective studies. Regression lines omit the intercept and two types of regressions are provided: unweighted and weighted by pooled standard errors from the two studies. The solid line indicates the identity line and the dotted line and dashed lines indicate unweighted and weighted regression, respectively. Only variants with *P* <1 × 10^−10^ in the reference study were included to mitigate the effect of the winner’s curse of inflated beta values in the reference studies.

Reporting Summary

Supplementary Data 1 List of variants, summary statistics and references for figures in the Supplementary Information file.

Supplementary Tables 1–12 Supplementary Tables 1–12 and table legends.

Supplementary Methods The file contains the following sections: phenotyping from nationwide population-based health registers; FinnGen participant recruitment and legacy cohorts, genotyping and genotype data QC; population structure and cryptic relatedness; GWAS and PheWAS analysis; and data access and dissemination. All supplementary figures are inlined in the appropriate sections.

Supplementary Note 1 Discussion of noteworthy findings from 15 previously well-studied benchmark diseases.

Supplementary Note 2 A list of all FinnGen working-group members and their affiliations.

## Data Availability

Based on National and European regulations (GDPR) access to individual-level sensitive health data must be approved by national authorities for specific research projects and for specifically listed and approved researchers. The health data described here was generated and provided by the National Health Register Authorities (Finnish Institute of Health and Welfare, Statistics Finland, KELA, Digital and Population Data Services Agency) and approved, either by the individual authorities or by the Finnish Data Authority, Findata, for use in the FinnGen project. Therefore, we, the authors of this paper, are not in a position to grant access to individual-level data to others. However, any researcher can apply for the health register data from the Finnish Data Authority Findata (https://findata.fi/en/permits/) and for individual-level genotype data from Finnish biobanks via the Fingenious portal (https://site.fingenious.fi/en/) hosted by the Finnish Biobank Cooperative FINBB (https://finbb.fi/en/). All Finnish biobanks can provide access for research projects within the scope regulated by the Finnish Biobank Act, which is research utilizing the biobank samples or data for the purposes of promoting health, understanding the mechanisms of disease or developing products and treatment practices used in health and medical care. The genotype data for the FinnGen release 5 used in this study was returned to the biobanks at the same time as the public release of the FinnGen release 5 summary results was done. All summary statistics described in this manuscript can be found in the Supplementary Information. All information regarding data download of summary statistics of additive GWAS of FinnGen release 5 can be found through the following link: https://finngen.gitbook.io/documentation/v/r5/data-download. You can learn more about accessing other FinnGen data here: https://www.finngen.fi/en/access_results. A full list of FinnGen end points for release 5 is available at: https://www.finngen.fi/en/researchers/clinical-endpoints. A full list of gene variants captured by the FinnGen specific Axiom array can be found at: https://www.finngen.fi/en/researchers/genotyping and https://www.dropbox.com/s/n8srnyy547resrq/finngen2_proposal_5_5_2019.tsv?dl=0.

## Code availability

Central data analysis and processing pipelines used are freely available: fine-mapping pipeline (https://github.com/FINNGEN/finemapping-pipeline); meta-analysis (https://github.com/FINNGEN/META_ANALYSIS); genetic ancestry and PCA pipeline (https://github.com/FINNGEN/pca_kinship); and GWAS SAIGE pipeline (https://github.com/FINNGEN/saige-pipelines). Please see https://finngen.gitbook.io/documentation/ for a detailed description of data production and analysis including code used to run analyses. Please see https://github.com/FINNGEN/ for further code repositories used to run analyses in FinnGen.

R v4.0.3 (https://www.r-project.org/) was used to create plots and analyse data. R codes used to reproduce figures are available upon request.

## References

[1] Lim ET (2014). Distribution and medical impact of loss-of-function variants in the Finnish founder population. PLoS Genet..

[2] Xue Y (2017). Enrichment of low-frequency functional variants revealed by whole-genome sequencing of multiple isolated European populations. Nat. Commun..

[3] Zuk, O. et al. Searching for missing heritability: designing rare variant association studies. *Proc. Natl Acad. Sci*. *USA*10.1073/pnas.1322563111 (2014). 10.1073/pnas.1322563111 PMC3910587 24443550

[4] Norio R (2003). The Finnish Disease Heritage III: the individual diseases. Hum. Genet..

[5] Auton A (2015). A global reference for human genetic variation. Nature.

[6] Bycroft C (2018). The UK Biobank resource with deep phenotyping and genomic data. Nature.

[7] Kerminen S (2017). Fine-scale genetic structure in Finland. G3.

[8] Ritari, J., Koskela, S., Hyvärinen, K., FinnGen & Partanen, J. HLA-disease association and pleiotropy landscape in over 235,000 Finns. *Hum. Immunol.* **83**, 391–398 (2022). 10.1016/j.humimm.2022.02.003 35221124

[9] Karczewski KJ (2020). The mutational constraint spectrum quantified from variation in 141,456 humans. Nature.

[10] Kanai, M. et al. Insights from complex trait fine-mapping across diverse populations. Preprint at *medRxiv*10.1101/2021.09.03.21262975 (2021).

[11] Sakaue S (2021). A cross-population atlas of genetic associations for 220 human phenotypes. Nat. Genet..

[12] Sun BB (2022). Genetic associations of protein-coding variants in human disease. Nature.

[13] Heyne, H. O. et al. Mono- and biallelic effects of on disease at biobank scale. *Nature*10.1038/s41586-022-05420-7 (2022). 10.1038/s41586-022-05420-7 PMC9849130 36653560

[14] Patel KA (2017). Heterozygous RFX6 protein truncating variants are associated with MODY with reduced penetrance. Nat. Commun..

[15] Norberg A (2018). Novel variants in Nordic patients referred for genetic testing of telomere-related disorders. Eur. J. Hum. Genet..

[16] Löf C (2016). Detection of novel gene variants associated with congenital hypothyroidism in a Finnish patient cohort. Thyroid.

[17] Jiang X-C, Yu Y (2021). The role of phospholipid transfer protein in the development of atherosclerosis. Curr. Atheroscler. Rep..

[18] Teslovich TM (2010). Biological, clinical, and population relevance of 95 loci for blood lipids. Nature.

[19] van der Harst P, Verweij N (2018). Identification of 64 novel genetic loci provides an expanded view on the genetic architecture of coronary artery disease. Circ. Res..

[20] Shadrina AS (2020). Prioritization of causal genes for coronary artery disease based on cumulative evidence from experimental and in silico studies. Sci. Rep..

[21] Dimitra A (2019). *HHIPL1*, a gene at the 14q32 coronary artery disease locus, positively regulates hedgehog signaling and promotes atherosclerosis. Circulation.

[22] Bindesbøll C (2020). NBEAL1 controls SREBP2 processing and cholesterol metabolism and is a susceptibility locus for coronary artery disease. Sci. Rep..

[23] Graydon, C. G., Mohideen, S. & Fowke, K. R. LAG3’s enigmatic mechanism of action. *Front. Immunol.*10.3389/fimmu.2020.615317 (2021). 10.3389/fimmu.2020.615317 PMC7820757 33488626

[24] No authors listed. (2022). FDA approves anti-LAG3 checkpoint. Nat. Biotechnol..

[25] Boer CG (2021). Deciphering osteoarthritis genetics across 826,690 individuals from 9 populations. Cell.

[26] Sliz E (2022). Uniting biobank resources reveals novel genetic pathways modulating susceptibility for atopic dermatitis. J. Allergy Clin. Immunol..

[27] Li J (2021). Utility of basophil activation test for predicting the outcome of wheezing in children: a pilot study. BMC Immunol..

[28] Chakraborti S, Natarajan K, Curiel J, Janke C, Liu J (2016). The emerging role of the tubulin code: from the tubulin molecule to neuronal function and disease. Cytoskeleton.

[29] Sharifinejad N (2020). Clinical, immunological, and genetic features in 49 patients with ZAP-70 deficiency: a systematic review. Front. Immunol..

[30] Mulford AJ, Wing C, Dolan ME, Wheeler HE (2021). Genetically regulated expression underlies cellular sensitivity to chemotherapy in diverse populations. Hum. Mol. Genet..

[31] Mafra F (2017). Copy number variation analysis reveals additional variants contributing to endometriosis development. J. Assist. Reprod. Genet..

[32] Koskela, J. T. et al. Genetic variant in *SPDL1* reveals novel mechanism linking pulmonary fibrosis risk and cancer protection. Preprint at *medRxiv*10.1101/2021.05.07.21255988 (2021).

[33] Ruotsalainen SE (2022). Inframe insertion and splice site variants in *MFGE8* associate with protection against coronary atherosclerosis. Commun. Biol..

[34] Rämö, J. T. et al. Genome-wide screen of otosclerosis in population biobanks: 27 loci and shared associations with skeletal structure. *Nat. Commun.*10.1038/s41467-022-32936-3 (2023). 10.1038/s41467-022-32936-3 PMC9849444 36653343

[35] Tanigawa Y (2020). Rare protein-altering variants in *ANGPTL7* lower intraocular pressure and protect against glaucoma. PLoS Genet..

[36] Surakka I (2015). The impact of low-frequency and rare variants on lipid levels. Nat. Genet..

[37] Ehrhardt N (2017). Hepatic Tm6sf2 overexpression affects cellular ApoB-trafficking, plasma lipid levels, hepatic steatosis and atherosclerosis. Hum. Mol. Genet..

[38] Prill S (2019). The *TM6SF2* E167K genetic variant induces lipid biosynthesis and reduces apolipoprotein B secretion in human hepatic 3D spheroids. Sci. Rep..

[39] Pirola CJ, Sookoian S (2015). The dual and opposite role of the *TM6SF2*-rs58542926 variant in protecting against cardiovascular disease and conferring risk for nonalcoholic fatty liver: a meta-analysis. Hepatology.

[40] Buch S (2015). A genome-wide association study confirms *PNPLA3* and identifies *TM6SF2* and *MBOAT7* as risk loci for alcohol-related cirrhosis. Nat. Genet..

[41] Tang S (2019). Association of *TM6SF2* rs58542926 T/C gene polymorphism with hepatocellular carcinoma: a meta-analysis. BMC Cancer.

[42] Kim DS (2017). Novel association of *TM6SF2* rs58542926 genotype with increased serum tyrosine levels and decreased apoB-100 particles in Finns. J. Lipid Res..

[43] Mars N (2020). Polygenic and clinical risk scores and their impact on age at onset and prediction of cardiometabolic diseases and common cancers. Nat. Med..

[44] Kiiskinen T (2020). Genomic prediction of alcohol-related morbidity and mortality. Transl Psychiatry.

[45] Strausz S (2021). Genetic analysis of obstructive sleep apnoea discovers a strong association with cardiometabolic health. Eur. Respir. J..

[46] Helkkula, P. et al. ANGPTL8 protein-truncating variant associated with lower serum triglycerides and risk of coronary disease. *PLoS Genet.* **17**, e1009501 (2021). 10.1371/journal.pgen.1009501 PMC8109807 33909604

[47] Rahimov, F. et al. High incidence and regional distribution of cleft palate in Finns are associated with a functional variant in an *IRF6* enhancer. Preprint at *Research Square*10.21203/rs.3.rs-941741/v1 (2021).

[48] Niemi, M. E. K. et al. Mapping the human genetic architecture of COVID-19. *Nature*10.1038/s41586-021-03767-x (2021). 10.1038/s41586-021-03767-x PMC8674144 34237774

[49] Zhou, W. et al. Global Biobank Meta-analysis Initiative: powering genetic discovery across human disease. *Cell Genom.* **2**, 100192 (2022). 10.1016/j.xgen.2022.100192 PMC9903716 36777996

[50] Broekema RV, Bakker OB, Jonkers IH (2020). A practical view of fine-mapping and gene prioritization in the post-genome-wide association era. Open Biol..

[51] Fry A (2017). Comparison of sociodemographic and health-related characteristics of UK Biobank participants with those of the general population. Am. J. Epidemiol..

[52] Pärn, K. et al. Genotyping chip data lift-over to reference genome build GRCh38/hg38 V.2. *protocols.io*10.17504/protocols.io.nqtddwn (2019).

[53] Palta, P. Genotype imputation workflow v3.0 V.1. *protocols.io*10.17504/protocols.io.nmndc5e (2018).

[54] Zhou W (2018). Efficiently controlling for case–control imbalance and sample relatedness in large-scale genetic association studies. Nat. Genet..

[55] Wang G, Sarkar A, Carbonetto P, Stephens M (2020). A simple new approach to variable selection in regression, with application to genetic fine mapping. J. R. Stat. Soc. Ser. B Stat. Methodol..

[56] Gagliano Taliun SA (2020). Exploring and visualizing large-scale genetic associations by using PheWeb. Nat. Genet..

[57] Mitt M (2017). Improved imputation accuracy of rare and low-frequency variants using population-specific high-coverage WGS-based imputation reference panel. Eur. J. Hum. Genet..

[58] *Pan-UK Biobank* (Pan UK Biobank Team, 2020); https://pan.ukbb.broadinstitute.org.

[59] Hail v.0.2 (Hail Team, 2019); https://github.com/hail-is/hail.

[60] Hormozdiari F (2016). Colocalization of GWAS and eQTL signals detects target genes. Am. J. Hum. Genet..

[61] Kerimov N (2021). A compendium of uniformly processed human gene expression and splicing quantitative trait loci. Nat. Genet..

[62] Ochoa D (2021). Open Targets Platform: supporting systematic drug–target identification and prioritisation. Nucleic Acids Res..

[63] Landrum MJ (2018). ClinVar: improving access to variant interpretations and supporting evidence. Nucleic Acids Res..

[64] Buniello A (2019). The NHGRI-EBI GWAS Catalog of published genome-wide association studies, targeted arrays and summary statistics 2019. Nucleic Acids Res..

