# Author Correction: FinnGen provides genetic insights from a well-phenotyped isolated population

**DOI:** 10.1038/s41586-023-05837-8

**Published:** 2023-02-24

**Authors:** Mitja I. Kurki, Juha Karjalainen, Priit Palta, Timo P. Sipilä, Kati Kristiansson, Kati M. Donner, Mary P. Reeve, Hannele Laivuori, Mervi Aavikko, Mari A. Kaunisto, Anu Loukola, Elisa Lahtela, Hannele Mattsson, Päivi Laiho, Pietro Della Briotta Parolo, Arto A. Lehisto, Masahiro Kanai, Nina Mars, Joel Rämö, Tuomo Kiiskinen, Henrike O. Heyne, Kumar Veerapen, Sina Rüeger, Susanna Lemmelä, Wei Zhou, Sanni Ruotsalainen, Kalle Pärn, Tero Hiekkalinna, Sami Koskelainen, Teemu Paajanen, Vincent Llorens, Javier Gracia-Tabuenca, Harri Siirtola, Kadri Reis, Abdelrahman G. Elnahas, Benjamin Sun, Christopher N. Foley, Katriina Aalto-Setälä, Kaur Alasoo, Mikko Arvas, Kirsi Auro, Shameek Biswas, Argyro Bizaki-Vallaskangas, Olli Carpen, Chia-Yen Chen, Oluwaseun A. Dada, Zhihao Ding, Margaret G. Ehm, Kari Eklund, Martti Färkkilä, Hilary Finucane, Andrea Ganna, Awaisa Ghazal, Robert R. Graham, Eric M. Green, Antti Hakanen, Marco Hautalahti, Åsa K. Hedman, Mikko Hiltunen, Reetta Hinttala, Iiris Hovatta, Xinli Hu, Adriana Huertas-Vazquez, Laura Huilaja, Julie Hunkapiller, Howard Jacob, Jan-Nygaard Jensen, Heikki Joensuu, Sally John, Valtteri Julkunen, Marc Jung, Juhani Junttila, Kai Kaarniranta, Mika Kähönen, Risto Kajanne, Lila Kallio, Reetta Kälviäinen, Jaakko Kaprio, FinnGen, Nurlan Kerimov, Johannes Kettunen, Elina Kilpeläinen, Terhi Kilpi, Katherine Klinger, Veli-Matti Kosma, Teijo Kuopio, Venla Kurra, Triin Laisk, Jari Laukkanen, Nathan Lawless, Aoxing Liu, Simonne Longerich, Reedik Mägi, Johanna Mäkelä, Antti Mäkitie, Anders Malarstig, Arto Mannermaa, Joseph Maranville, Athena Matakidou, Tuomo Meretoja, Sahar V. Mozaffari, Mari E. K. Niemi, Marianna Niemi, Teemu Niiranen, Christopher J. O´Donnell, Ma´en Obeidat, George Okafo, Hanna M. Ollila, Antti Palomäki, Tuula Palotie, Jukka Partanen, Dirk S. Paul, Margit Pelkonen, Rion K. Pendergrass, Slavé Petrovski, Anne Pitkäranta, Adam Platt, David Pulford, Eero Punkka, Pirkko Pussinen, Neha Raghavan, Fedik Rahimov, Deepak Rajpal, Nicole A. Renaud, Bridget Riley-Gillis, Rodosthenis Rodosthenous, Elmo Saarentaus, Aino Salminen, Eveliina Salminen, Veikko Salomaa, Johanna Schleutker, Raisa Serpi, Huei-yi Shen, Richard Siegel, Kaisa Silander, Sanna Siltanen, Sirpa Soini, Hilkka Soininen, Jae Hoon Sul, Ioanna Tachmazidou, Kaisa Tasanen, Pentti Tienari, Sanna Toppila-Salmi, Taru Tukiainen, Tiinamaija Tuomi, Joni A. Turunen, Jacob C. Ulirsch, Felix Vaura, Petri Virolainen, Jeffrey Waring, Dawn Waterworth, Robert Yang, Mari Nelis, Anu Reigo, Andres Metspalu, Lili Milani, Tõnu Esko, Caroline Fox, Aki S. Havulinna, Markus Perola, Samuli Ripatti, Anu Jalanko, Tarja Laitinen, Tomi P. Mäkelä, Robert Plenge, Mark McCarthy, Heiko Runz, Mark J. Daly, Aarno Palotie

**Affiliations:** 1 Institute for Molecular Medicine Finland (FIMM), Helsinki Institute of Life Science (HiLIFE), University of Helsinki, Helsinki, Finland; 2 Program in Medical and Population Genetics, Broad Institute of Harvard and MIT, Cambridge, MA USA; 3 Stanley Center for Psychiatric Research, Broad Institute of Harvard and MIT, Cambridge, MA USA; 4 Analytic and Translational Genetics Unit, Massachusetts General Hospital, Boston, MA USA; 5 Estonian Genome Centre, Institute of Genomics, University of Tartu, Tartu, Estonia; 6 Finnish Institute for Health and Welfare (THL), Helsinki, Finland; 7 Medical and Clinical Genetics, University of Helsinki and Helsinki University Hospital, Helsinki, Finland; 8 Department of Obstetrics and Gynecology, Tampere University Hospital, Tampere, Finland; 9 Faculty of Medicine and Health Technology, Center for Child, Adolescent and Maternal Health, University of Tampere, Tampere, Finland; 10 Helsinki Biobank, University of Helsinki and Hospital District of Helsinki and Uusimaa, Helsinki, Finland; 11 Department of Biomedical Informatics, Harvard Medical School, Boston, MA USA; 12 Digital Health Center, Hasso Plattner Institute for Digital Engineering, University of Potsdam Potsdam, Potsdam, Germany; 13 Hasso Plattner Institute for Digital Health at Mount Sinai, Department of Genetics and Genomic Sciences, Icahn School of Medicine at Mount Sinai, New York, NY USA; 14 TAUCHI Research Center, Faculty of Information Technology and Communication Sciences, Tampere University, Tampere, Finland; 15 Translational Biology, Research and Development, Biogen, Cambridge, MA USA; 16 BHF Cardiovascular Epidemiology Unit, Department of Public Health and Primary Care, University of Cambridge, Cambridge, UK; 17 Optima Partners, Edinburgh, UK; 18 MRC Biostatistics Unit, School of Clinical Medicine, University of Cambridge, Cambridge, UK; 19 Faculty of Medicine and Health Technology, Tampere University, Tampere, Finland; 20 Institute of Computer Science, University of Tartu, Tartu, Estonia; 21 Finnish Red Cross Blood Service, Helsinki, Finland; 22 GlaxoSmithKline, Espoo, Finland; 23 Bristol Myers Squibb, New York, NY USA; 24 Tampere University Hospital and Tampere University, Tampere, Finland; 25 Biogen, Cambridge, MA USA; 26 Boehringer Ingelheim, Ingelheim am Rhein, Germany; 27 GlaxoSmithKline, Collegeville, PA USA; 28 Division of Rheumatology, Department of Medicine, Helsinki University Central Hospital, Helsinki, Finland; 29 Orton Orthopedic Hospital, Helsinki, Finland; 30 Abdominal Center, Helsinki University Hospital, Helsinki University, Helsinki, Finland; 31 Maze Therapeutics, South San Francisco, CA USA; 32 Auria Biobank, University of Turku and Turku University Hospital, Turku, Finland; 33 FINBB, Finnish Biobank Cooperative, Helsinki, Finland; 34 Pfizer, New York, NY USA; 35 Department of Medicine, Karolinska Institute, Solna, Sweden; 36 Clinical Biobank Tampere, Tampere University and Tampere University Hospital, Tampere, Finland; 37 Medical Research Center Oulu and PEDEGO Research Unit, University of Oulu, Oulu, Finland; 38 Biocenter Oulu, University of Oulu, Oulu, Finland; 39 Oulu University Hospital, Oulu, Finland; 40 Department of Psychology and Logopedics, Faculty of Medicine, University of Helsinki, Helsinki, Finland; 41 SleepWell Research Program, Faculty of Medicine, University of Helsinki, Helsinki, Finland; 42 Merck & Co, Kenilworth, NJ USA; 43 PEDEGO Research Unit, University of Oulu, Oulu, Finland; 44 Department of Dermatology and Medical Research Center Oulu, Oulu University Hospital, Oulu, Finland; 45 Genentech, San Francisco, CA USA; 46 AbbVie, Chicago, IL USA; 47 Helsinki University Hospital and University of Helsinki, Helsinki, Finland; 48 Neuro Center, Neurology, Kuopio University Hospital, Kuopio, Finland; 49 Institute of Clinical Medicine–Neurology, University of Eastern Finland, Kuopio, Finland; 50 Northern Finland Biobank Borealis, University of Oulu, Northern Ostrobothnia Hospital District, Oulu, Finland; 51 Department of Ophthalmology, Kuopio University Hospital, Kuopio, Finland; 52 Department of Ophthalmology, Institute of Clinical Medicine, University of Eastern Finland, Kuopio, Finland; 53 Department of Clinical Physiology, Tampere University Hospital, Tampere, Finland; 54 Epilepsy Center, Kuopio University Hospital, Kuopio, Finland; 55 Department of Neurology, University of Eastern Finland, Kuopio, Finland; 56 Department of Public Health, University of Helsinki, Helsinki, Finland; 57 Computational Medicine, Center for Life Course Health Research, Faculty of Medicine, University of Oulu, Oulu, Finland; 58 Translational Sciences, Sanofi R&D, Framingham, MA USA; 59 Biobank of Eastern Finland, University of Eastern Finland, Kuopio, Finland; 60 Kuopio University Hospital, Kuopio, Finland; 61 Central Finland Biobank, Central Finland Health Care District, Jyväskylä, Finland; 62 Department of Clinical Genetics, Tampere University Hospital, Tampere, Finland; 63 Department of Clinical Genetics, Faculty of Medicine and Health Technology, Tampere, Finland; 64 Department of Medicine, Institute of Clinical Medicine, University of Eastern Finland, Kuopio, Finland; 65 FINBB, Finnish Biobank Cooperative, Turku, Finland; 66 Department of Otorhinolaryngology–Head and Neck Surgery, University of Helsinki, Helsinki, Finland; 67 Helsinki University Hospital, Helsinki, Finland; 68 Pfizer, Cambridge, MA USA; 69 Department of Medical Epidemiology and Biostatistics, Karolinska Institute, Solna, Sweden; 70 Centre for Genomics Research, Discovery Sciences, BioPharmaceuticals R&D, AstraZeneca, Cambridge, UK; 71 TAUCHI Research Center & Faculty of Medicine and Health Technology, Tampere University, Tampere, Finland; 72 Turku University Hospital and University of Turku, Turku, Finland; 73 Novartis Institutes for BioMedical Research, Cambridge, MA USA; 74 Anesthesia, Critical Care, and Pain Medicine, Massachusetts General Hospital, Boston, MA USA; 75 Department of Oral and Maxillofacial Diseases, Helsinki University Hospital, Helsinki, Finland; 76 Department of Oral and Maxillofacial Diseases, University of Helsinki, Helsinki, Finland; 77 Finnish Hematological Biobank, Helsinki, Finland; 78 Department of Pulmonary Diseases, Kuopio University Hospital, Kuopio, Finland; 79 Department of Otorhinolaryngology, Helsinki University Hospital and University of Helsinki, Helsinki, Finland; 80 Translational Science and Experimental Medicine, Research and Early Development, Respiratory and Immunology, BioPharmaceuticals R&D, AstraZeneca, Cambridge, UK; 81 GlaxoSmithKline, Stevenage, UK; 82 Department of Clinical Genetics, HUSLAB, HUS Diagnostic Center, University of Helsinki, Helsinki, Finland; 83 Novartis Institutes for BioMedical Research, Basel, Switzerland; 84 Finnish Clinical Biobank Tampere, Tampere University and Tampere University Hospital, Tampere, Finland; 85 Department of Neurology, Institute of Clinical Medicine, University of Eastern Finland, Kuopio, Finland; 86 Department of Neurology, Helsinki University Hospital, Helsinki, Finland; 87 Translational Immunology, Research Programs Unit, University of Helsinki, Helsinki, Finland; 88 Department of Allergy, Helsinki University Hospital and University of Helsinki, Helsinki, Finland; 89 Abdominal Center, Endocrinology, Helsinki University Hospital, Helsinki, Finland; 90 Folkhalsan Research Center, Helsinki, Finland; 91 Research Program of Clinical and Molecular Metabolism, University of Helsinki, Helsinki, Finland; 92 Eye Genetics Group, Folkhälsan Research Center, Helsinki, Finland; 93 University of Turku, Turku, Finland; 94 Janssen Research & Development, Spring House, PA USA; 95 Janssen Biotech, Beerse, Belgium; 96 Genomics Core Facility, Institute of Genomics, University of Tartu, Tartu, Estonia; 97 Helsinki Institute of Life Science (HiLIFE), University of Helsinki, Helsinki, Finland

**Keywords:** Genome-wide association studies, Genetics research, Genetic predisposition to disease, Rare variants

Correction to: *Nature* 10.1038/s41586-022-05473-8 Published online 18 January 2023

In the version of this article initially published, author Marianna Niemi, now affiliated with the TAUCHI Research Center and the Faculty of Medicine and Health Technology in Tampere University, was mistakenly affiliated with TAUCHI Research Center within the Faculty of Information Technology and Communication Sciences department. The error has been corrected in the HTML and PDF versions of the article.

