# Supplementary material for: FinnGen provides genetic insights from a well-phenotyped isolated population
Source: Nature. 2023 Jan 18;613(7944):508–18. doi: 10.1038/s41586-022-05473-8 (PMC9849126; doi:10.1038/s41586-022-05473-8)
Supplement: Supplementary file 1 — Comparison of effects size in known genome-wide significant loci between FinnGen and large published reference GWASs (Table 1). The y and x axes represent FinnGen and reference GWAS effect sizes, respectively. Beta values are aligned to be positive in reference studies. Lines extending from points indicate standard errors in respective studies. Regression lines omit the intercept and two types of regressions are provided: unweighted and weighted by pooled standard errors from the two studies. The solid line indicates the identity line and the dotted line and dashed lines indicate unweighted and weighted regression, respectively. Only variants with P <1 × 10^−10^ in the reference study were included to mitigate the effect of the winner’s curse of inflated beta values in the reference studies. [file 41586_2022_5473_MOESM1_ESM.pdf]

nature portfolio

https://doi.org/10.1038/s41586-022-05473-8

Supplementary information

FinnGen provides genetic insights from a well-phenotyped isolated population

In the format provided by the authors and unedited

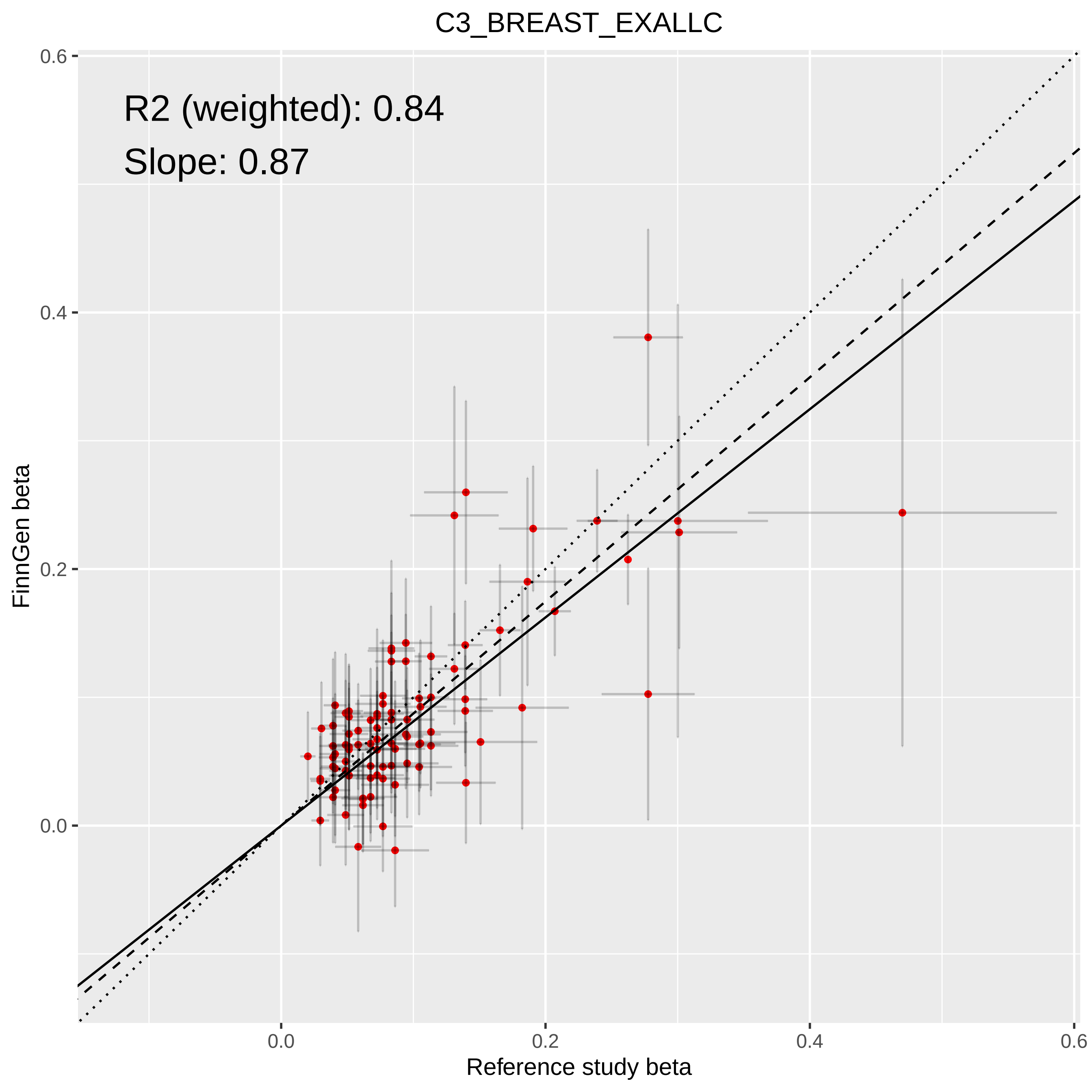
C3_BREAST_EXALLC
R2 (weighted): 0.84
Slope: 0.87
FinnGen beta
Reference study beta
0.6
0.4
0.2
0.0
0.0
0.2
0.4
0.6

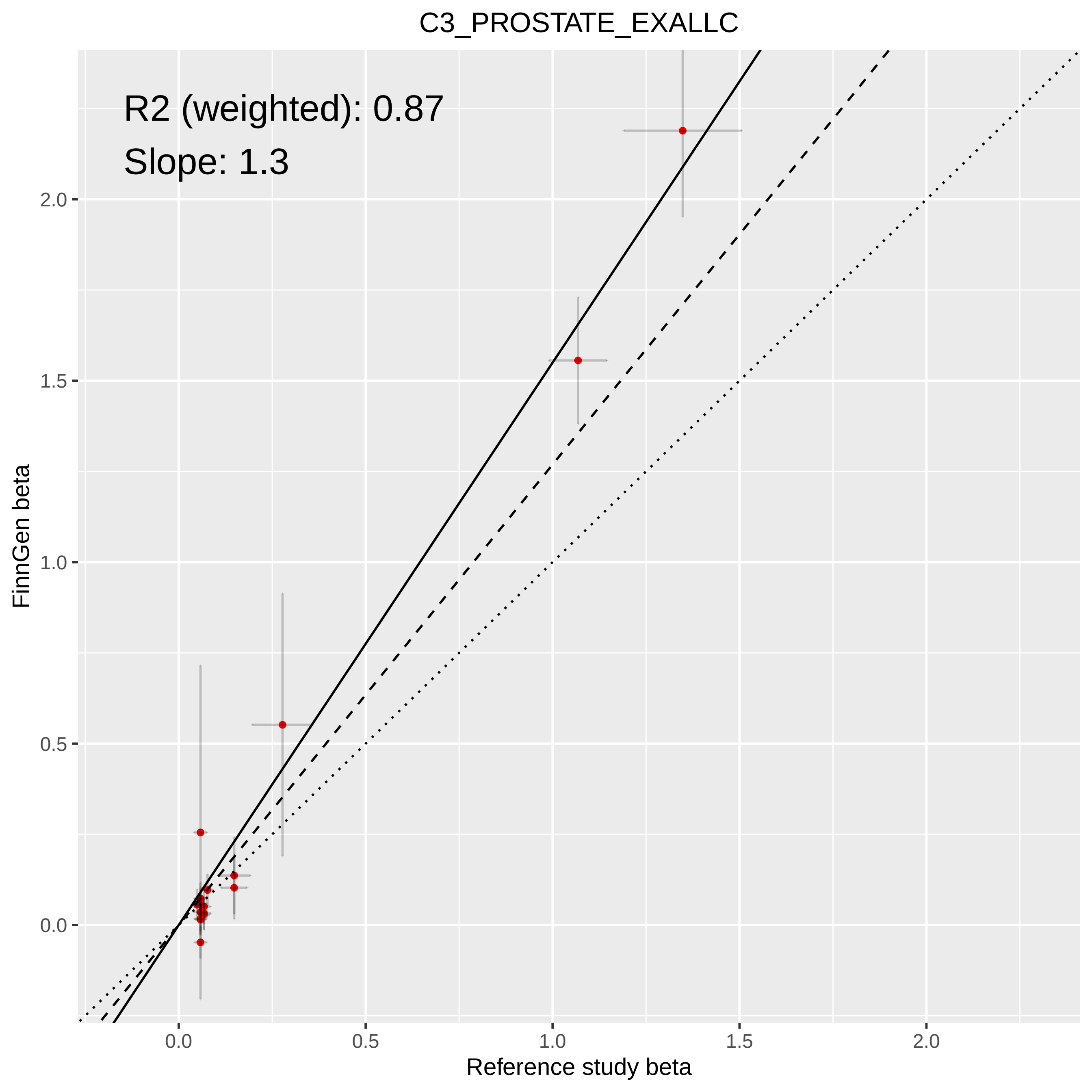
C3_PROSTATE_EXALLC
R2 (weighted): 0.87
Slope: 1.3
FinnGen beta
Reference study beta
0.0
0.5
1.0
1.5
2.0
0.0
0.5
1.0
1.5
2.0

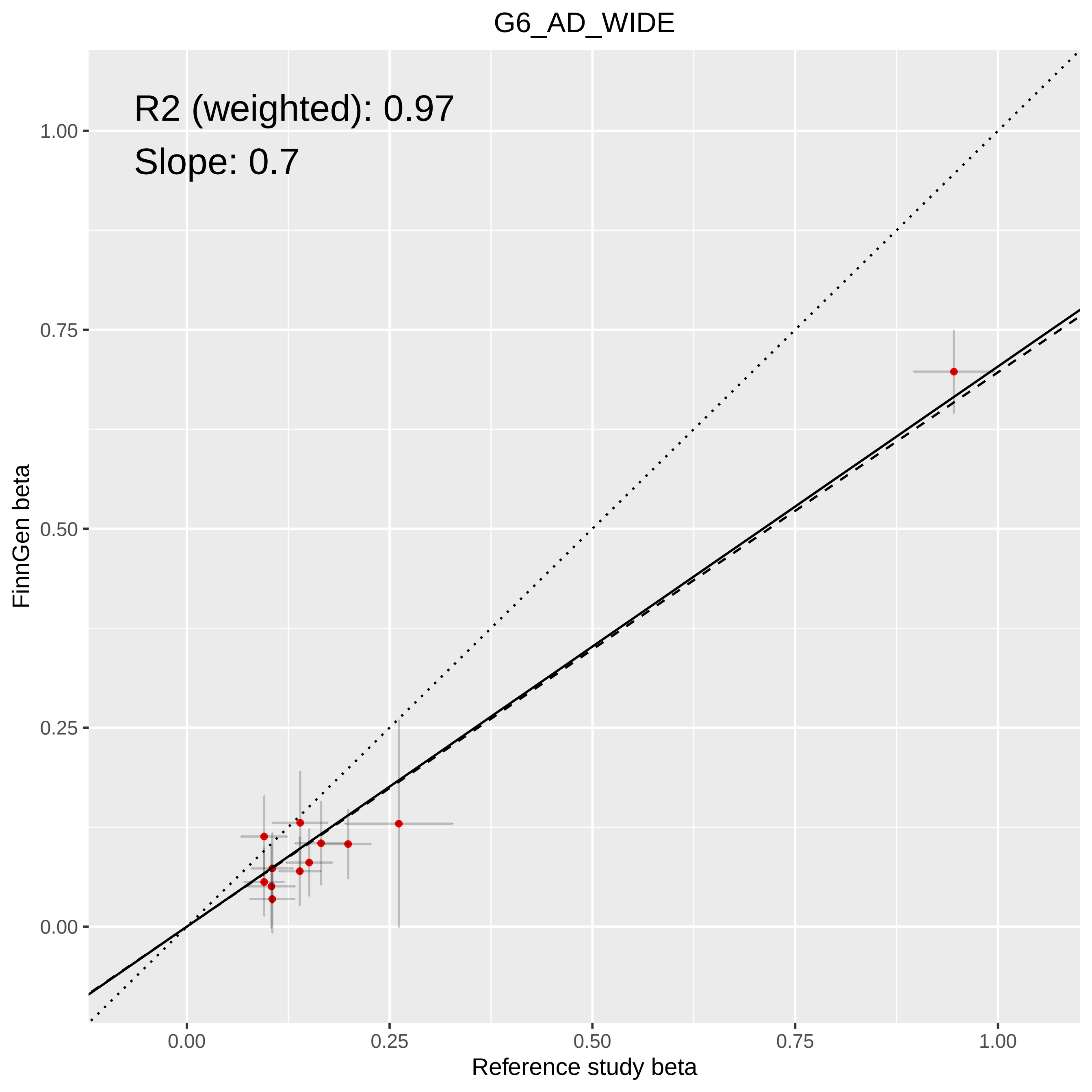

G6_AD_WIDE
R2 (weighted): 0.97
Slope: 0.7
FinnGen beta
Reference study beta
0.00
0.25
0.50
0.75
1.00
0.00
0.25
0.50
0.75
1.00

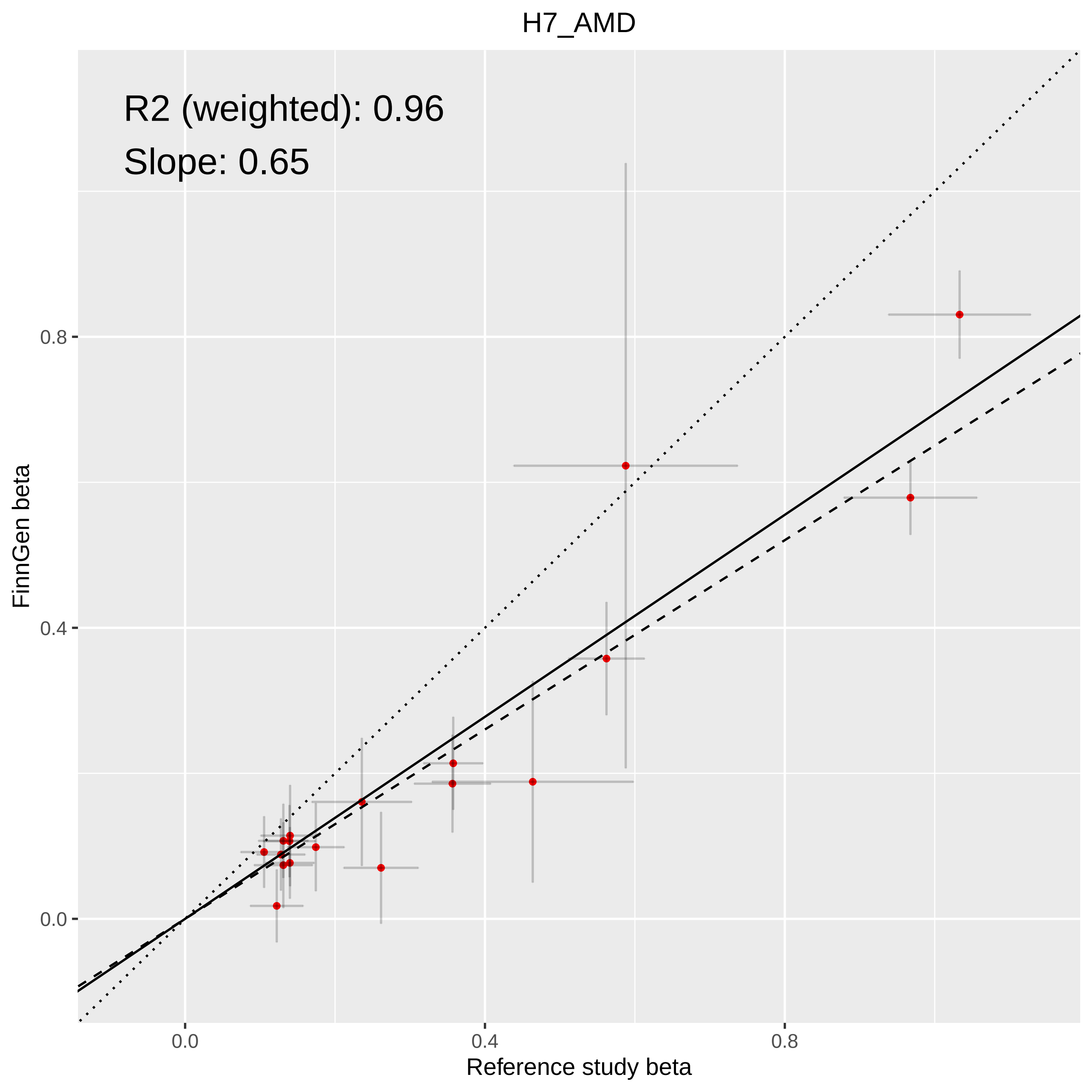
H7_AMD
R2 (weighted): 0.96
Slope: 0.65
FinnGen beta
Reference study beta
0.0
0.4
0.8
0.0
0.4
0.8

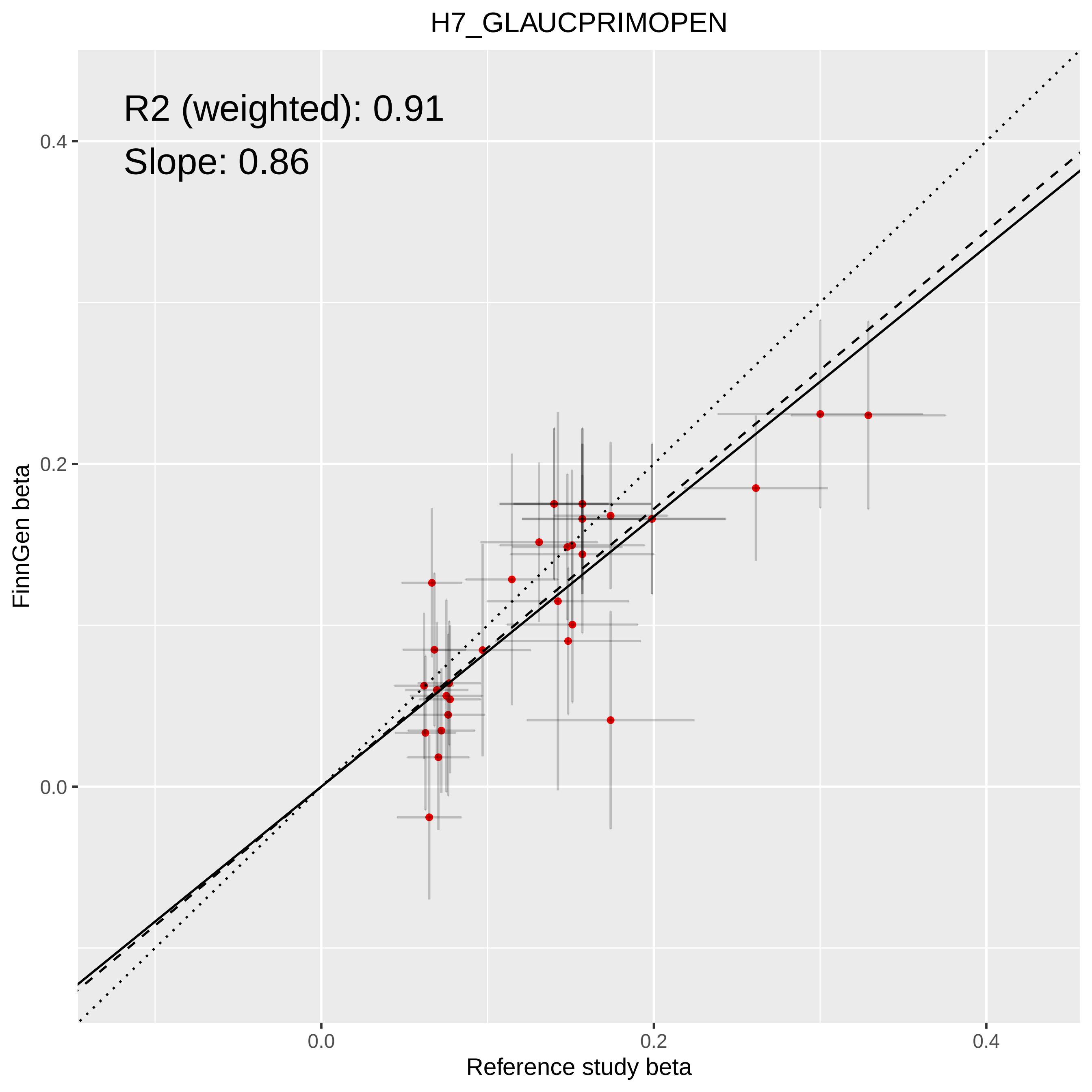
H7_GLAUCPRIMOPEN
R2 (weighted): 0.91
Slope: 0.86
FinnGen beta
Reference study beta
0.4
0.2
0.0
0.0
0.2
0.4

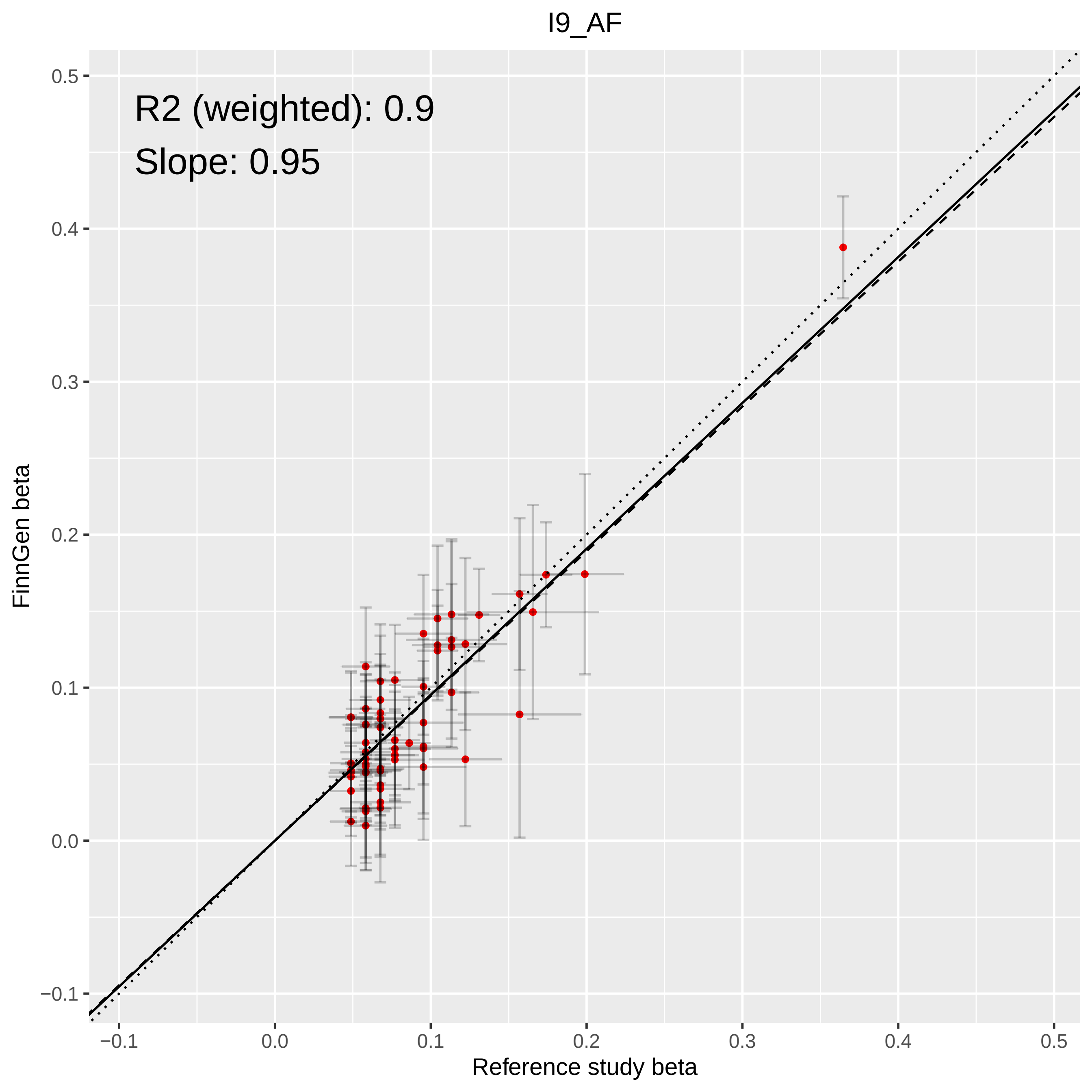
I9_AF
R2 (weighted): 0.9
Slope: 0.95
FinnGen beta
Reference study beta
0.5
0.4
0.3
0.2
0.1
0.0
−0.1
−0.1
0.0
0.1
0.2
0.3
0.4
0.5

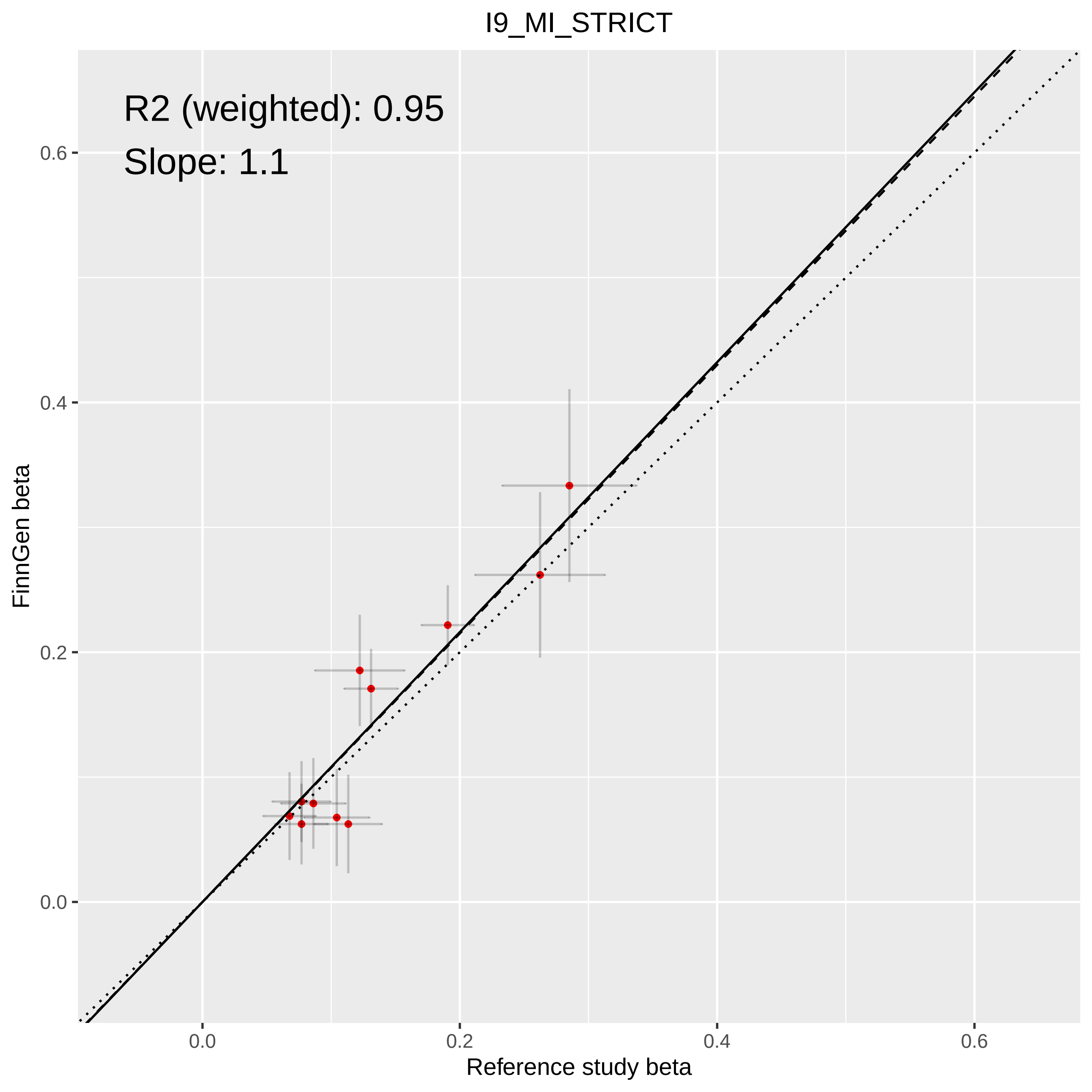

I9_MI_STRICT
R2 (weighted): 0.95
Slope: 1.1
FinnGen beta
Reference study beta
0.0
0.2
0.4
0.6
0.0
0.2
0.4
0.6

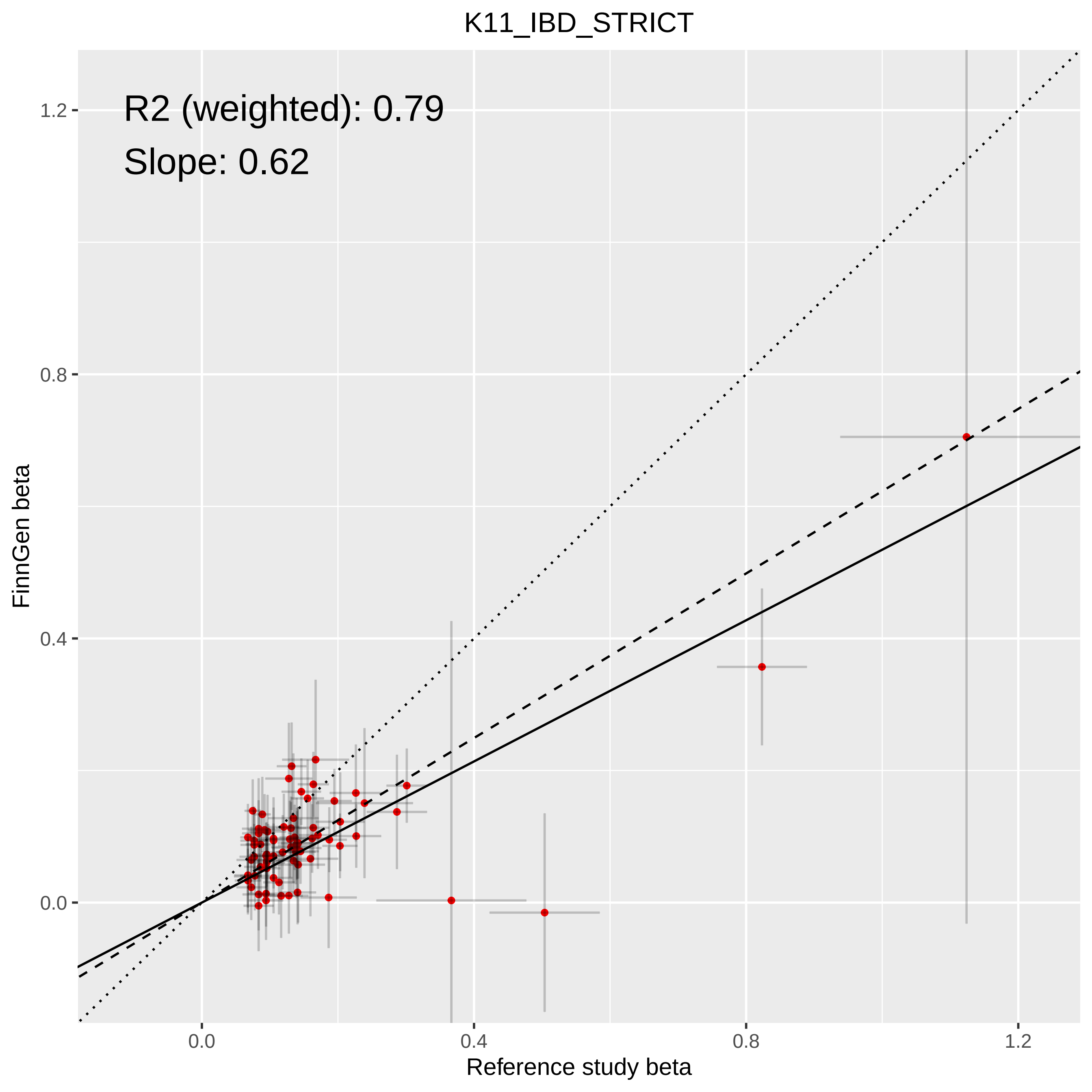
K11_IBD_STRICT
R2 (weighted): 0.79
Slope: 0.62
FinnGen beta
Reference study beta
0.0
0.4
0.8
1.2

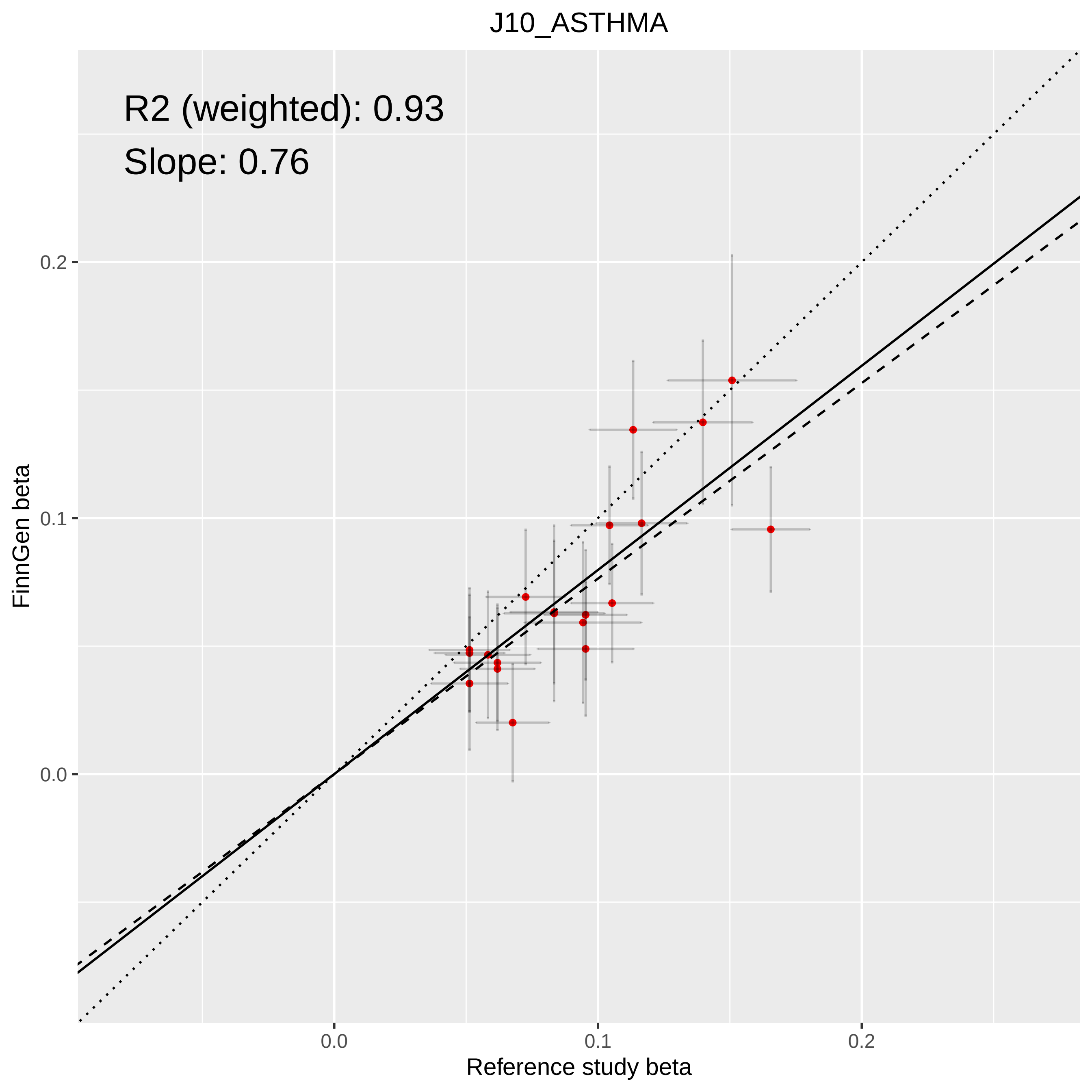

J10_ASTHMA
R2 (weighted): 0.93
Slope: 0.76
FinnGen beta
Reference study beta
0.2
0.1
0.0
0.0
0.1
0.2

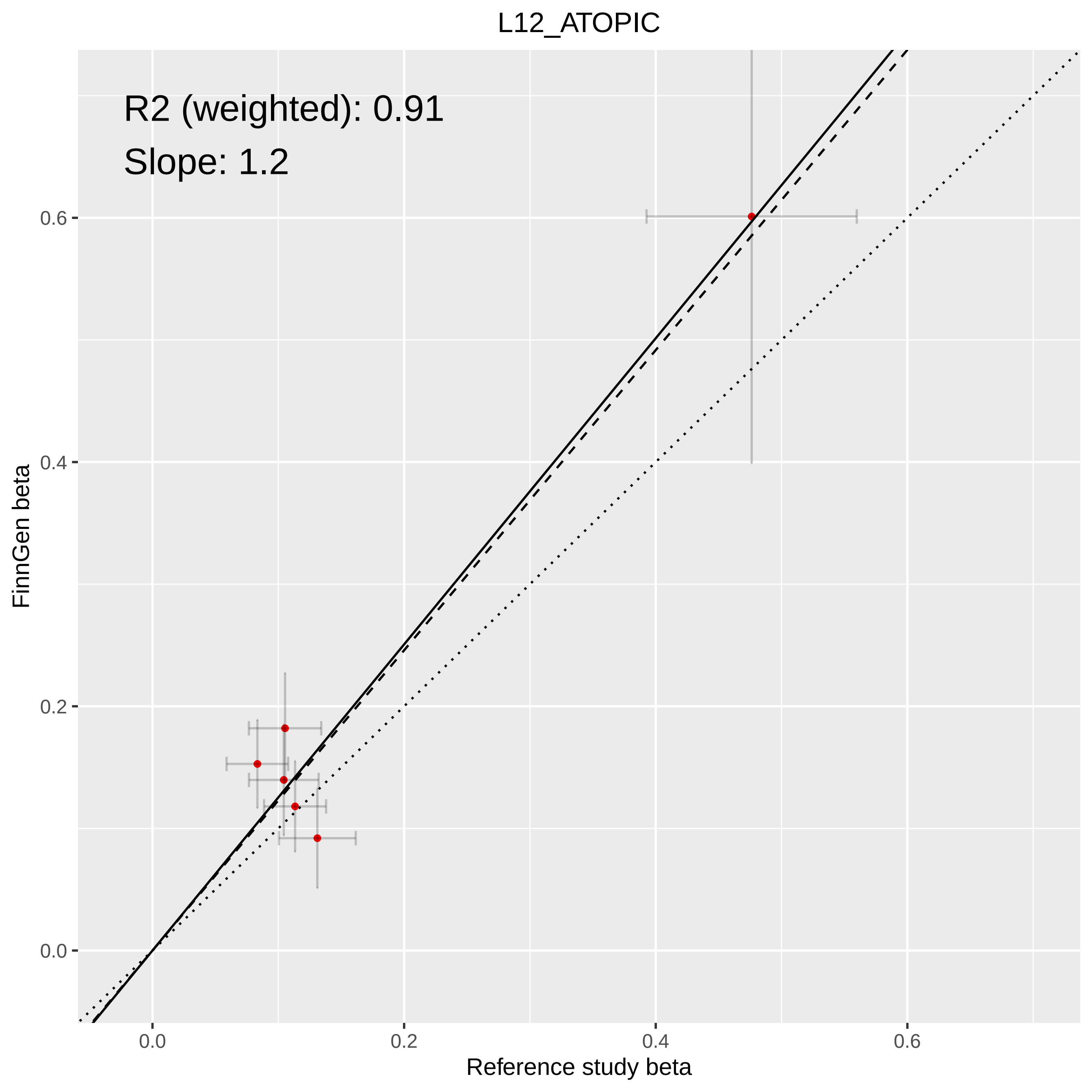
L12_ATOPIC
R2 (weighted): 0.91
Slope: 1.2
0.6
0.4
0.2
0.0
FinnGen beta
0.0
0.2
0.4
0.6
Reference study beta

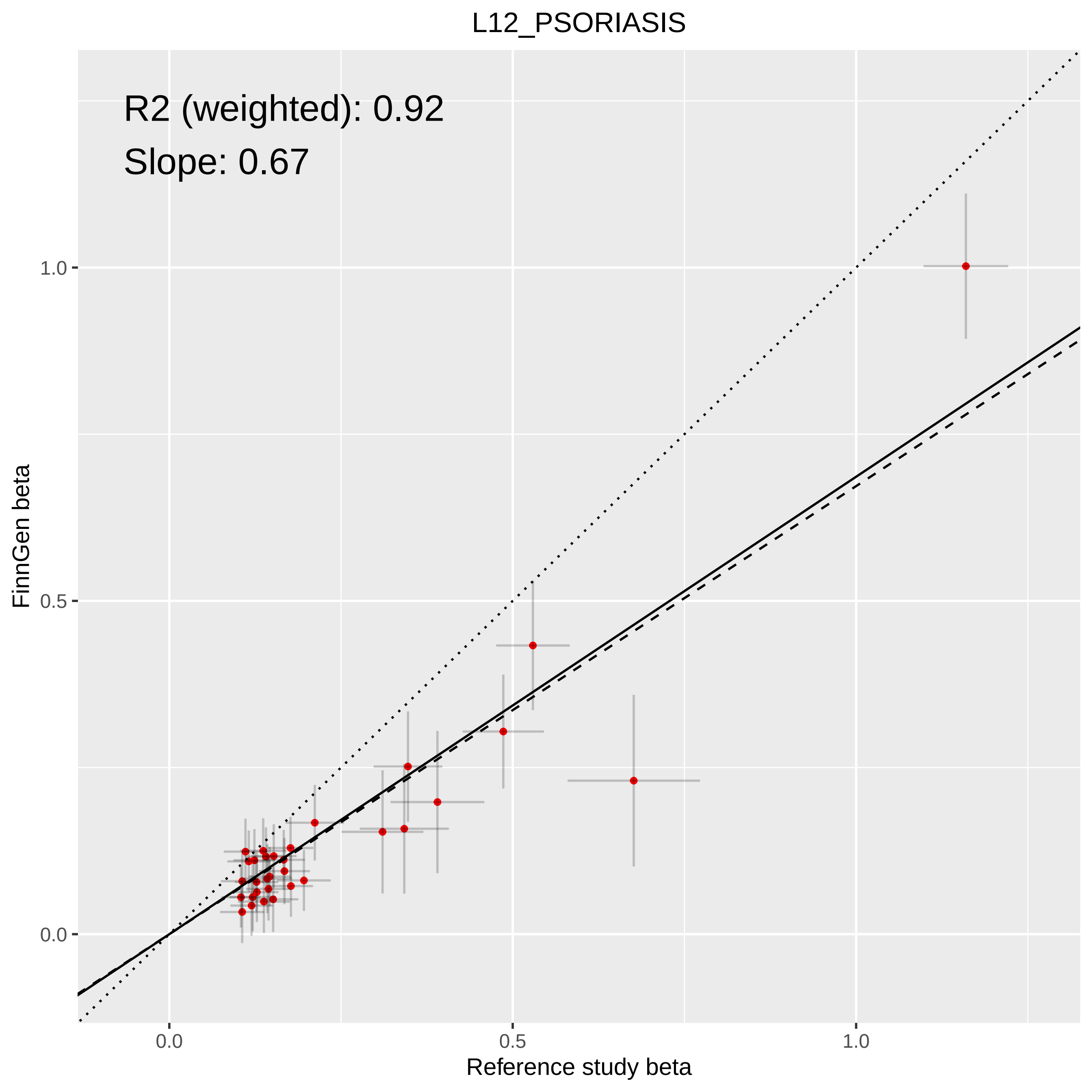

L12_PSORIASIS
R2 (weighted): 0.92
Slope: 0.67
FinnGen beta
Reference study beta
0.0
0.5
1.0
0.0
0.5
1.0

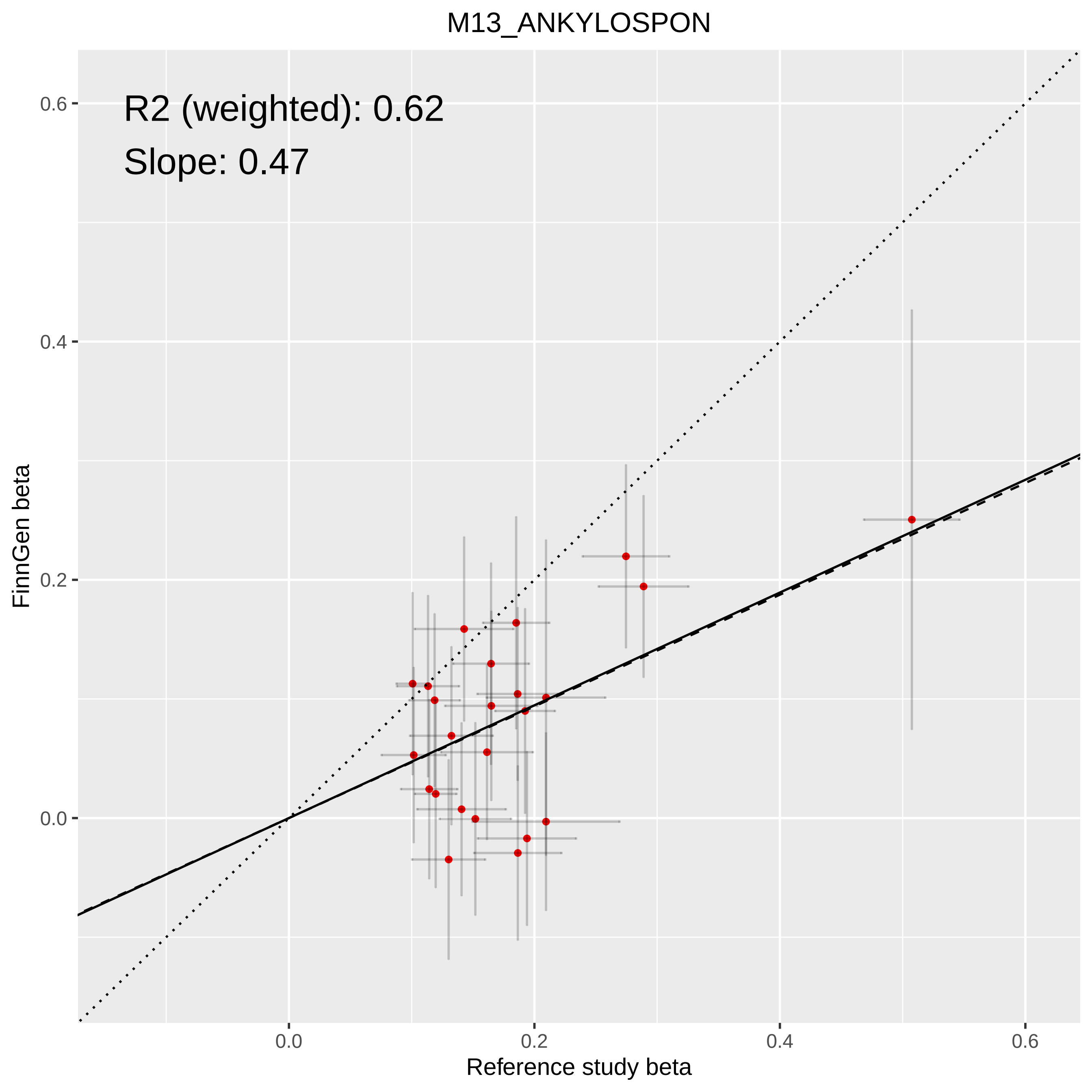

M13_ANKYLOSPON
R2 (weighted): 0.62
Slope: 0.47
FinnGen beta
Reference study beta
0.6
0.4
0.2
0.0
0.0
0.2
0.4
0.6

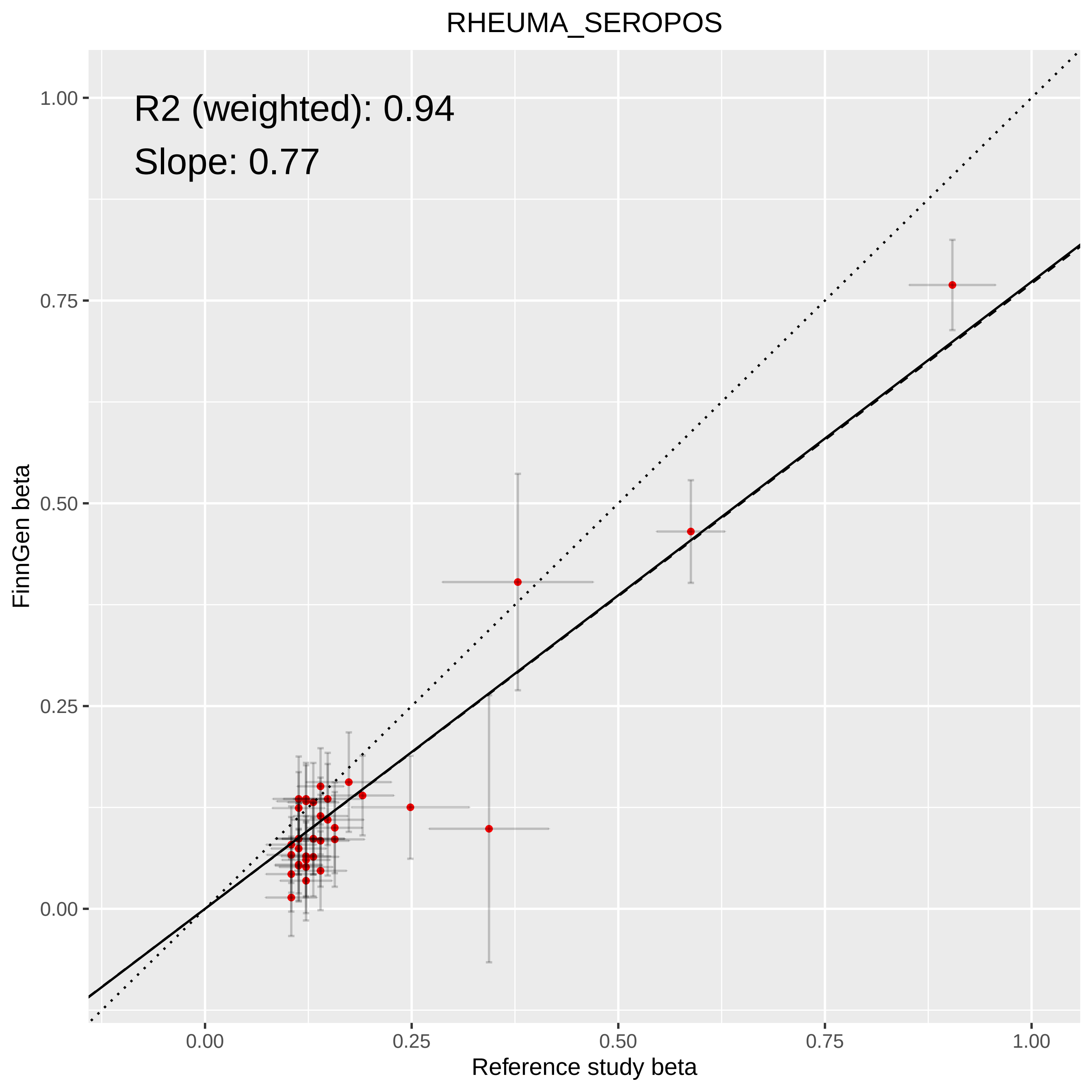
RHEUMA_SEROPOS
R2 (weighted): 0.94
Slope: 0.77
FinnGen beta
Reference study beta
0.00
0.25
0.50
0.75
1.00
0.00
0.25
0.50
0.75
1.00

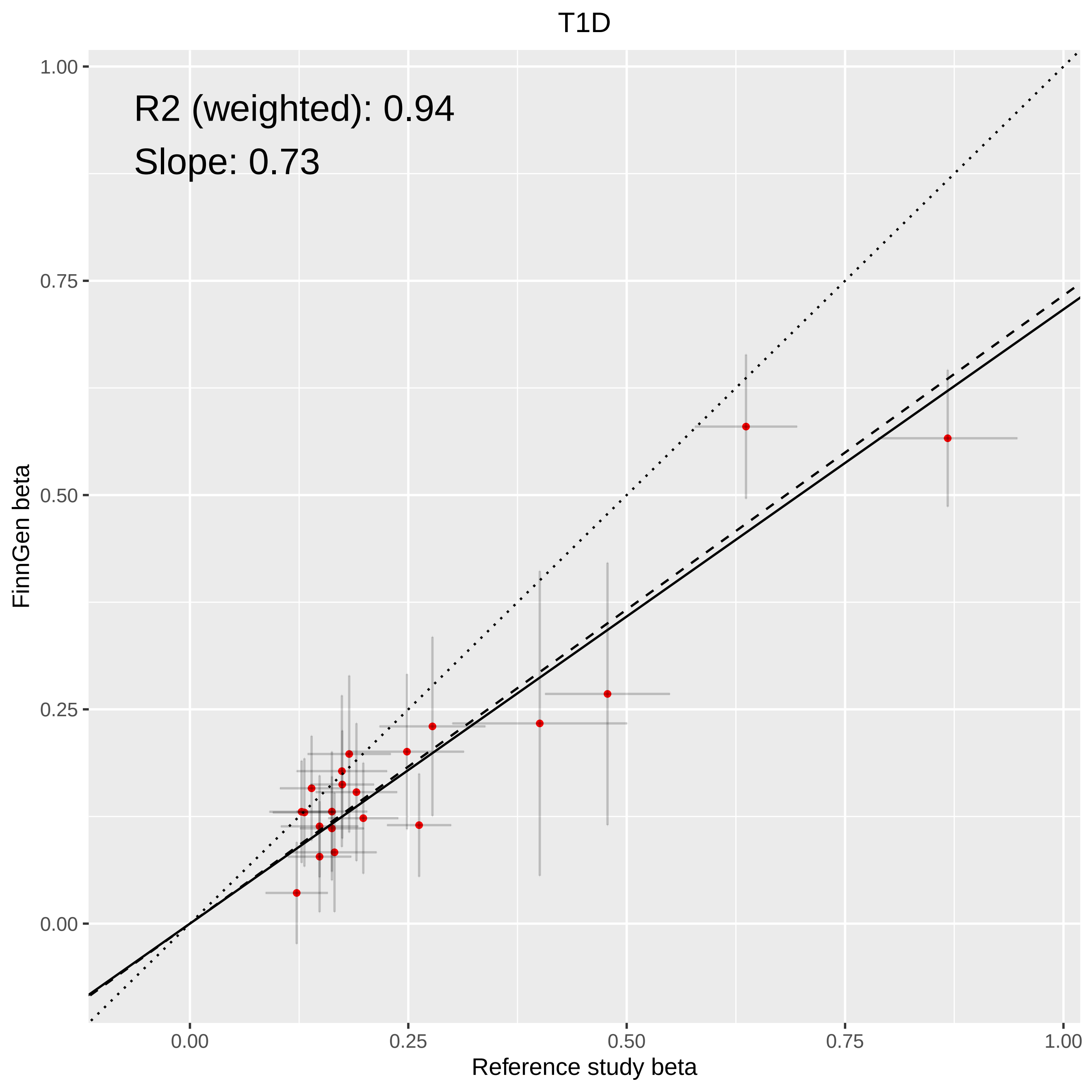

T1D
R2 (weighted): 0.94
Slope: 0.73
FinnGen beta
Reference study beta
1.00
0.75
0.50
0.25
0.00
0.00
0.25
0.50
0.75
1.00

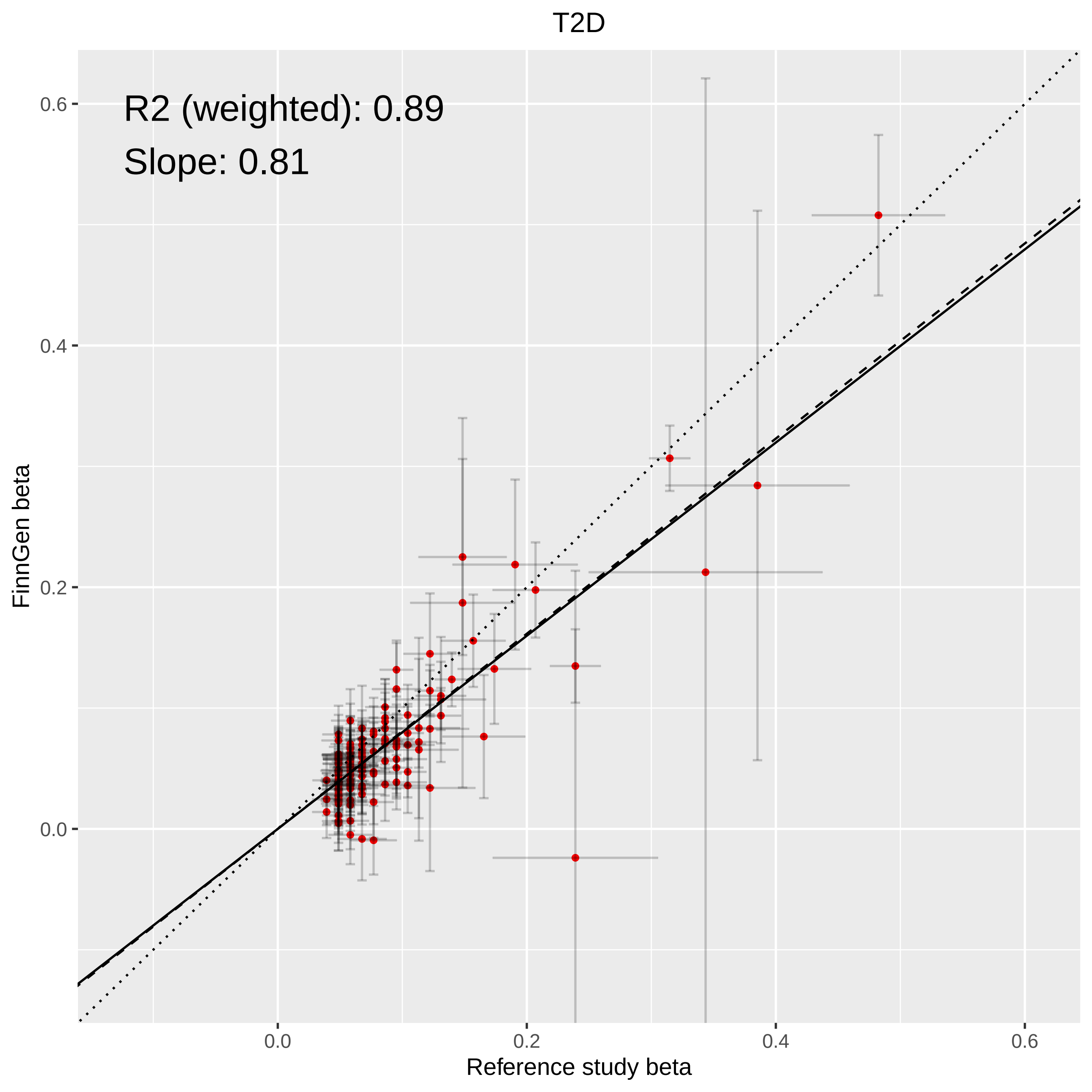
T2D
R2 (weighted): 0.89
Slope: 0.81
FinnGen beta
Reference study beta
0.0
0.2
0.4
0.6
0.0
0.2
0.4
0.6
