# Supplementary material for: FinnGen provides genetic insights from a well-phenotyped isolated population
Source: Nature. 2023 Jan 18;613(7944):508–18. doi: 10.1038/s41586-022-05473-8 (PMC9849126; doi:10.1038/s41586-022-05473-8)
Supplement: Supplementary file 6 — Discussion of noteworthy findings from 15 previously well-studied benchmark diseases. [file 41586_2022_5473_MOESM6_ESM.docx]

Supplementary Note 1

Noteworthy novel findings from 15 previously well-studied benchmark diseases

**Age-related macular degeneration**

A Finnish enriched 3’ UTR variant in CFI was associated with age-related macular degeneration (chr4_109740713_T_A, FinnGen OR 1.92. P: 1.8*10-7). The variant has been observed almost exclusively in Finnish population (gnomAD AF 1.3%) but exists at a very low frequency in Estonian (gnomAD AF 0.02%) and `other` populations in gnomAD (AF 0.008%). Remarkably, the association was replicated in Estonian biobank (AF 0.05%, OR 6.75, p: 2.19*10-3) despite it being rare in Estonia. Consistent with the variant consequence, we observed an eQTL colocalization with strong gene expression lowering effect of CFI gene transcript in adipose tissue (CLPP 0.46, beta -1.6) in a FUSION transcriptome^1^ study using Finnish samples. CFI codes for complement factor I, a regulator of classical and alternative complement pathways. Rare recessive missense and stop gained variants in CFI have been observed in complement factor I deficiency and, consistent with the eQTL directionality here, heterozygous carriers of such reduced function variants have been strongly associated with AMD in exome sequencing of AMD GWAS loci^2^.

**Atrial fibrillation**

Four novel low frequency (MAF 0.1%-2%) associations were observed for atrial fibrillation and flutter. In all four we identified a putative causal coding variant in credible sets. Two independent coding variants (chr16_1947063_G_A,chr16_1954141_C_T) were observed in RPL3L. Both variant associations were supported by Estonian and UKBB replication. Coding variants in RPL3L that are not in LD (r2<0.005 to lead variants in FinnGen imputation panel) with our associations have been previously associated with atrial fibrillation. In a GWAS meta-analysis with 14,710 cases and 373,897 controls from Iceland and 14,792 cases and 393,863 controls from the UK Biobank, a missense and splice donor variants in RPL3L were associated with atrial fibrillation^3^.

The third atrial fibrillation association lead variant (chr19_50497261_C_T, AF 2%, OR 1.43, p 1.92 * 10-12) was 16.6 times enriched in Finland. The variant exists in the Estonian population and the effect size was consistent although markedly lower and not statistically significant ( AF 0.6%, OR 1.16, p 0.46). The variant did not exist in UKBB data (North-Western europeans in GnomAD AF 0.1%). We observed a missense variant (p.Arg1845Trp) in MYH14 in the credible set, as well as eQTL colocation with upregulation of putative long non-coding RNA (ENSG00000268518) in skeletal muscle in the Finnish FUSION study1. Variants in MYH14 can cause autosomal dominant peripheral neuropathy and deafness (https://www.omim.org/entry/608568) but no cardiac phenotype has been previously reported.

In the fourth association (AF 1.3%, OR 1.5, p 8.17*10-11) we observed a splice donor variant (c.105+1G>T) in the SYNPO2L gene (PIP 0.13). The variant was extremely rare in Estonia (0.05%) but showed consistent effect direction (OR 1.23) although not significant due to lack of power in such rare variant association. The variant was absent in UKBB and the AF in NFSEE in gnomad is extremely low (0.02%). An intronic common variant in SYNPO2L has been previously associated with atrial fibrillation^4^ Our results provide direct coding variant evidence of SYNPO2L being the causal gene in this locus. Further insights on atrial fibrillation from FinnGen coding variants have been described by Sun et al^5^.

Prostate cancer

Two novel associations were identified for prostate cancer. The first was a 16-fold Finnish-enriched variant ( chr22_43082544_C_T, AF 0.3%, OR 3.49, p 5.44*10-8) and showed consistent effect size in Estonia (AF 0.09%, OR 3, p 0.2) but was absent in UKBB (meta-analysis p 2.45*10-8). An inframe deletion variant (p.Ala139_Leu148del) in BIK gene was observed in the credible set (PIP 0.44). Common variants in the locus have been previously associated with prostate cancer^6^. BIK (BCL-2 interacting killer) gene product is a pro-apoptotic protein that has been suggested to act as a tumor suppressor gene^7^ and to be a marker for a more aggressive breast cancer^8^. Somatic prostate cancer mutations in BIK have been observed, however no direct evidence of inherited coding variation has previously been reported as associated with prostate cancer.

The other prostate cancer association (chr17_42937117_C_G, OR 7.1 p 1.21 * 10-8, MAF 0.1%) was a very rare variant with only 3 copies observed in gnomAD (2 in Finns and 1 in ‘Other’ gnomAd population). Replication of the association was not possible due to the variant’s rarity in other populations and replication in an independent cohort is warranted. The lead variant (PIP 0.62) is a non-coding variant downstream from SNORA40 and the other equally rare (3 copies in Finns and 1 in ‘Other’ populations in gnomAD) variant (chr17_42372576_G_C, rs1202093388, MAF 0.09%, OR 9.4, 5.1*10-8, PIP 0.37) in 95% credible set resides 600kb away in an intron of STAT3. SNORA40 codes for small nucleolar RNA (SNORA), the role of which in cancer are not very well characterized but differential expression associations of several SNORAs with different cancers have been observed^9^. Hyperactivation of STAT3 however has been observed in the majority of cancers and their prognosis including prostate cancer^10^. STAT3 is a transcription factor activated by IL6/JAK/STAT3-pathway and mediates many inflammatory processes. Chronic inflammation can induce development and progression of tumors^10^. Protein-truncating and missense variants in STAT3 are depleted in human populations ( PTV observed/expected upper bound 0.1, missense observed/expected upped bound 0.36; gnomAD v2.1.111). No effect on survival was observed in cases only survival analysis from diagnosis to death (hazard ratio 1.04, p 0.94).

Asthma

Another interesting locus where we observed coding variants (missense in IL4R, p.Ala82Thr, PIP 0.21) within credible sets was associated with both asthma (FinnGen AF 8.2%, OR 0.86, p 2.5*10-12, meta p 5.31*10-9 ) and psoriasis (FinnGen OR 1.28, p 3.48*10-9, meta p 1.92*10-11) but with an opposing direction of effect. IL4R codes for IL4α subunit that is part of receptor complexes for both cytokines IL4 and IL13, which are key cytokines in the type ​​II inflammatory response triggered by allergens or parasites (see^12^ for a detailed review). The key role of type II inflammatory response in asthma has long been recognized13 and variants in 5q31 locus containing the genes coding for IL4 and IL13 have been associated with asthma in GWA studies^14^. Asthma, atopic dermatitis and hay fever often co-occur and are referred collectively to as atopic diseases^15^. The effect direction of this association was consistent with that of asthma in atopic dermatitis (OR 0.9, p 2.4 * 10-3). The reversed effect direction in psoriasis was surprising as there is no evidence that in psoriasis there would be a contribution of type II inflammation but type | and Th17 mediated inflammation^16^.

1. Taylor, D. L. *et al.* Integrative analysis of gene expression, DNA methylation, physiological traits, and genetic variation in human skeletal muscle. *Proc. Natl. Acad. Sci.* **116**, 10883–10888 (2019).

2. Seddon, J. M. *et al.* Rare variants in CFI , C3 and C9 are associated with high risk of advanced age-related macular degeneration. *Nat. Genet.* **45**, 1366–1370 (2013).

3. Thorolfsdottir, R. B. *et al.* Coding variants in RPL3L and MYZAP increase risk of atrial fibrillation. *Commun. Biol.* **1**, 1–9 (2018).

4. Roselli, C. *et al.* Multi-ethnic genome-wide association study for atrial fibrillation. *Nat. Genet.* **50**, 1225–1233 (2018).

5. Sun, B. B. *et al.* Genetic associations of protein-coding variants in human disease. *Nature* **603**, 95–102 (2022).

6. Eeles, R. A. *et al.* Identification of seven new prostate cancer susceptibility loci through a genome-wide association study. *Nat. Genet.* **41**, 1116–1121 (2009).

7. Chinnadurai, G., Vijayalingam, S. & Rashmi, R. BIK, the founding member of the BH3-only family proteins: mechanisms of cell death and role in cancer and pathogenic processes. *Oncogene* **27**, S20–S29 (2008).

8. Pandya, V. *et al.* BIK drives an aggressive breast cancer phenotype through sublethal apoptosis and predicts poor prognosis of ER-positive breast cancer. *Cell Death Dis.* **11**, 1–19 (2020).

9. Deogharia, M. & Majumder, M. Guide snoRNAs: Drivers or Passengers in Human Disease? *Biology* **8**, (2018).

10. Abdulghani, J. *et al.* Stat3 Promotes Metastatic Progression of Prostate Cancer. *Am. J. Pathol.* **172**, 1717–1728 (2008).

11. Karczewski, K. J. *et al.* The mutational constraint spectrum quantified from variation in 141,456 humans. *Nature* **581**, 434–443 (2020).

12. Junttila, I. S. Tuning the Cytokine Responses: An Update on Interleukin (IL)-4 and IL-13 Receptor Complexes. *Front. Immunol.* **9**, (2018).

13. Lambrecht, B. N., Hammad, H. & Fahy, J. V. The Cytokines of Asthma. *Immunity* **50**, 975–991 (2019).

14. Demenais, F. *et al.* Multiancestry association study identifies new asthma risk loci that colocalize with immune-cell enhancer marks. *Nat. Genet.* **50**, 42–53 (2018).

15. Thomsen, S. F. Epidemiology and natural history of atopic diseases. *Eur. Clin. Respir. J.* **2**, (2015).

16. Boehncke, W.-H. & Schön, M. P. Psoriasis. *Lancet Lond. Engl.* **386**, 983–994 (2015).
